# Macrophage‐Hepatocyte Circuits Mediated by Grancalcin Aggravate the Progression of Metabolic Dysfunction Associated Steatohepatitis

**DOI:** 10.1002/advs.202406500

**Published:** 2024-09-16

**Authors:** Tian Su, Yue He, Min Wang, Haiyan Zhou, Yan Huang, Mingsheng Ye, Qi Guo, Ye Xiao, Guangping Cai, Mingyang Zhao, Jianping Wang, Xianghang Luo

**Affiliations:** ^1^ Department of Endocrinology Endocrinology Research Center Xiangya Hospital of Central South University Changsha Hunan 410008 China; ^2^ Department of Endocrinology The Second Affiliated Hospital of University of South China Hengyang Hunan 421000 China; ^3^ National Clinical Research Center for Geriatric Disorders Xiangya Hospital Changsha Hunan 410008 China

**Keywords:** GCA, macrophage‐hepatocyte, metabolic dysfunction associated steatohepatitis

## Abstract

The dynamic interplay between parenchymal hepatocytes and non‐parenchymal cells (NPCs), such as macrophages, is an important mechanism for liver metabolic homeostasis. Although numerous endeavors have been made to identify the mediators of metabolic dysfunction associated steatohepatitis (MASH), the molecular underpinnings of MASH progression remain poorly understood, and therapies to arrest MASH progression remain elusive. Herein, it is revealed that the expression of grancalcin (GCA) is upregulated in the macrophages of patients and rodents with MASH and correlates with MASH progression. Notably, the administration of recombinant GCA aggravates the development of MASH, whereas, *Gca* deletion in myeloid cells blunts liver steatosis and inflammation in multiple MASH murine models. Mechanistically, GCA activates macrophages via TLR9‐NF‐κB signaling, and the activated macrophages promote hepatocyte lipid accumulation and apoptosis via secretion of Interleukin‐6(IL‐6), Tumor Necrosis Factor α (TNFα), and Interleukin‐1β(IL‐1β), thereby leading to hepatic steatosis and inflammation. Finally, the therapeutic administration of antibody blocking GCA effectively halts the progression of MASH. Collectively, these findings implicate GCA as a crucial mediator of MASH and clarify a new metabolic signaling axis between the hepatocytes and macrophages, implying that GCA can emerge as a particularly interesting putative therapeutic target for reversing MASH progression.

## Graphical abstract

The interplay between macrophages and hepatocyte remains largely unknown. Herin, a highly significant function of macrophage‐hepatocyte communication is described in which the upregulation of GCA is involved in the progression of MASH. The findings greatly extend the current understanding of the pathophysiological role of macrophages in liver metabolism and suggest that GCA may be a potential treatment targets for MASH.

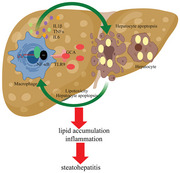

## 1 Introduction

In 2023, three multinational liver associations proposed the term “metabolic dysfunction‐associated steatotic liver disease” (MASLD) to replace non‐alcoholic fatty liver disease (NAFLD).^[^ ^1^ ^]^ Notably, the new nomenclature for fatty liver disease emphasizes the concept of metabolic dysfunction associated with the condition and highlights the necessity of assessing individual characteristics of metabolic syndrome.^[^ ^2^ ^]^ Moreover, there is a consensus that research data from previous studies on NAFLD/ non‐alcoholic steatohepatitis (NASH) can still be referenced and utilized following the adoption of this new MASLD terminology.^[^ ^3^ ^]^ Parallel to the increasing prevalence of obesity and diabetes, MASLD has become the most common chronic liver disease worldwide, affecting ≈25% of the global population.^[^ ^4^ ^]^ Metabolic dysfunction associated steatohepatitis (MASH), characterized by hepatic steatosis, inflammation, chronic liver injury, and fibrosis, is a severe form of MASLD.^[^ ^5^ ^]^ MASH is emerging as a major risk factor for cirrhosis, liver failure, and hepatocellular cancer and is a leading cause of liver transplantation.^[^ ^6^ ^]^ Although numerous endeavors have been made to identify the mediators of MASH, the molecular underpinnings of MASH progression remain poorly understood, and therapies to arrest MASH progression remain elusive.

Interactions and communication between parenchymal hepatocytes and non‐parenchymal cells (NPCs), including immune cells, hepatic stellate cells (HSCs), and liver sinusoidal endothelial cells (ECs), are important for maintaining adequate liver metabolic homeostasis. Previous research has indicated that Treg cell‐derived Areg promotes the activation of quiescent HSCs and that activated HSCs promote hepatocyte gluconeogenesis via Interleukin‐6 (IL‐6) in MASH.^[^ ^7^ ^]^ The signal exchange between hepatocytes and macrophages is particularly prominent in liver metabolic homeostasis. Macrophage‐derived Osteopontin (SPP1) promoted fatty acid oxidation (FAO) in hepatocytes, thereby alleviating hepatic steatosis.^[^ ^8^ ^]^ Macrophage X‐box binding protein‐1(XBP1) induces lipid metabolism disorders and proinflammatory cytokine expression in hepatocytes via activation of NLR Family Pyrin Domain Containing 3 (NLRP3) inflammasome.^[^ ^9^ ^]^ Whereas DNA and excess free fatty acids in hepatocytes are secreted into the surrounding macrophages to activate the macrophage inflammatory pathway.^[^ ^10^ ^]^ In our previous study, we identified a proinflammation factor grancalcin (GCA), released by mono‐macrophages, which induces skeletal aging.^[^ ^11^ ^]^ GCA is a secreted protein that is particularly abundant in macrophages and neutrophils and belongs to the penta‐EF‐hand protein family. It plays a vital role in multiple biological functions, including the regulation of host defense,^[^ ^12^ ^]^ cell migration,^[^ ^13^ ^]^ activation of autophagy,^[^ ^14^ ^]^ and inflammatory signals.^[^ ^11^ ^]^ Our previous study found that myeloid derived GCA instigated obesity‐induced insulin resistance and metabolic inflammation.^[^ ^15^ ^]^ As obesity significantly increases the risk of developing MASLD and ≈80% of patients with MASLD are obese,^[^ ^16^ ^]^ this prompted us to conjecture the unique biological significance of GCA in MASLD.

Herein, we reveal that the expression of GCA is upregulated in macrophages from patients and rodents with MASH and correlates with MASH progression. Notably, administration with recombinant GCA aggravates the development of MASH, whereas, *Gca* deletion in myeloid cells blunts liver steatosis and inflammation in multiple murine models of MASH. Mechanistically, GCA activates macrophages via TLR9(Toll Like Receptor 9)‐NF‐κB(nuclear factor kappa‐B) signaling, and activated macrophages promote hepatocyte lipid accumulation and apoptosis via secretion of IL‐6, Tumor Necrosis Factor α (TNFα) and Interleukin‐1β(IL‐1β), thereby leading to hepatic steatosis and inflammation. Finally, the therapeutic administration of an antibody blocking GCA effectively halts the progression of MASH. Collectively, these findings implicate GCA as crucial mediator of MASH and clarify a new metabolic signaling axis between the hepatocytes and macrophages, which may raise the possibility that GCA could emerge as a particularly interesting putative therapeutic target to reverse MASH progression.

## 2 Results

### 2.1 GCA Expression is Upregulated in Macrophages of Patients and Rodents with MASH and Correlates with MASH Progression

**Figure 1:**
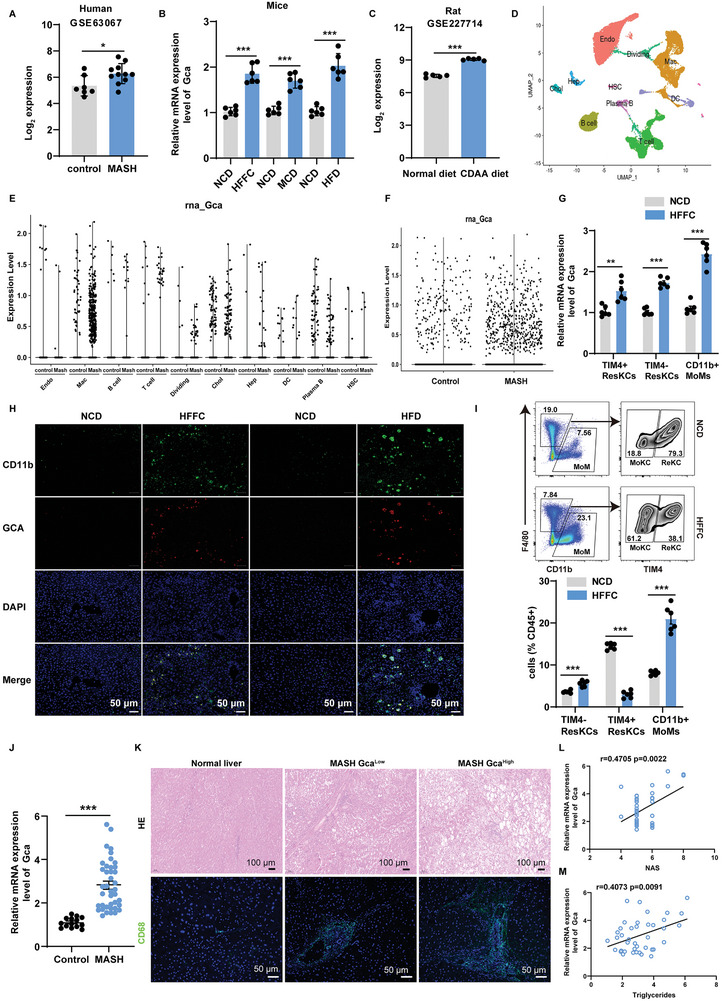
The expression of GCA is upregulated in macrophages from patients and rodents with MASH and correlates with MASH progression. A) Liver *Gca* expression among patients with metabolic dysfunction associated steatohepatitis (MASH) (n = 11) or healthy controls (n = 7) as determined by microarray data. B) *Gca* mRNA expression in murine livers as assessed by qPCR (n = 6 per group). C) Liver *Gca* expression among rats fed with choline‐deficient L‐amino acid‐defined (CDAA) diet or normal chow diet (NCD) as determined by microarray data (n = 5). D) UMAP visualization of liver cell clusters based on 33168 single cell transcriptomes. Cell counts for endothelial cells (Endo), macrophages, T cells, B cells, dendritic cells (DC), cholangiocytes (Chol), hepatocytes (Hep), dividing cells, plasma B cells and hepatic stellate cells (HSC) are indicated in parentheses. E) Violin plots of log‐transformed gene expression of *Gca* genes in cell populations of livers from control mice (NCD) and MASH (amylin diet, AMLN diet) mice. F) Violin plots of log‐transformed gene expression of *Gca* genes in livers from control mice (NCD) and MASH (amylin diet, AMLN diet) mice. G) Expression of *Gca* in hepatic CD45^+^Ly6g^−^F4/80^+^TIM4^+^ ResKC, CD45^+^Ly6g^−^F4/80^+^TIM4^−^ MoKC and CD45^+^Ly6g^−^F4/80^−^CD11b^+^ MoMs sorted from the livers of mice fed with normal chow diet (NCD) or high‐fat, fructose, and cholesterol (HFFC) diet for 24 weeks (n = 6 per group). H) Representative immunofluorescence staining showing the expression of GCA (red) and CD11b (green) in the mouse liver (n = 6 per group). Scale bars: 50 µm. I) Flow cytometry analysis of CD45^+^Ly6g^−^F4/80^+^TIM4^+^ ResKC, CD45^+^Ly6g^−^F4/80^+^TIM4‐ MoKC, and CD45^+^Ly6g^−^F4/80^−^CD11b^+^ MoMs in the livers of mice fed NCD or HFFC diet (n = 6 per group). J) *Gca* mRNA levels in the liver tissues of MASH patients (n = 40) compared with normal controls (n = 14), as determined by qPCR. K) Representative images of HE (scale bar, 100 µm) and immunofluorescence staining of CD68 (scale bar, 50 µm) in the liver tissues of MASH patients compared with normal controls. L) Graphs show the correlation between the mRNA levels of hepatic *Gca* and NAS in patients with MASH (n = 40). M) Graphs show the correlation between the mRNA levels of hepatic *Gca* and triglyceride in patients with MASH (n = 40). Data were shown as mean ± SEM. Statistical analysis was assessed by two‐sided Student's t test (A‐C, G, I, J) or two‐sided Spearman's correlation (L, M). *p < 0.05, **p < 0.01, ***p < 0.001.

In our previous study, we found that expression levels of GCA were elevated in both obese mice and human participants.^[^ ^15^ ^]^ Since MASLD/MASH is an obesity associated metabolic disease, we hypothesized that GCA may have detrimental effects on MASH progression. To examine the expression of *GCA* in liver, we analyzed mRNA expression arrays from a dataset (GSE63067) containing human liver biopsies of healthy controls and patients with MASH. The results revealed that compared with healthy controls, *GCA* mRNA expression was significantly elevated in patients with MASH (**Figure** **1**A).

Moreover, we detected the expression of *Gca* in the livers of mouse models of MASH, including high‐fat, fructose, and cholesterol (HFFC) diet‐induced MASH, methionine‐ and choline‐deficient (MCD) diet‐induced MASH and high fat diet (HFD)‐induced MASH. Consistent with human studies, the levels of *Gca* mRNA were also significantly increased in the liver tissues obtained from all models (Figure 1B). In line with the findings in human and mouse models, bioinformatics analysis of gene expression in rat liver tissue (GSE227714 and GSE65220) also validated the elevation of *Gca* mRNA levels in MASH (Figure 1C; Figure S1A, Supporting Information).

In order to pinpoint the cellular source of increased hepatic *Gca* expression during MASH, we performed bioinformatic analysis using a single‐cell RNA sequencing (scRNA‐seq, GSE129516) dataset from healthy and MASH mouse livers. We identified 10 major clusters, including ECs, macrophages, T cells, B cells, cholangiocytes, plasma B cells, dendritic cells (DC), HSCs, hepatocytes, and a cluster representing dividing cells, according to the marker gene expression (Figure 1D). We found that *Gca* was highly expressed in macrophages and nearly non‐expressed in hepatocytes of MASH livers (Figure 1E). Moreover, compared to the chow‐diet livers, the number of GCA^+^ cells was higher in MASH livers (Figure 1F).

In the mouse liver, there are three primary types of macrophage populations: TIM4^+^((T‐cell immunoglobulin and mucin domain‐containing 4) resident Kupffer cells (ResKCs), TIM4^−^ monocyte‐derived Kupffer cells (MoKCs), and CD11b^+^ monocyte‐derived macrophages (MoMs).^[^ ^17^ ^]^ Therefore, we assessed *Gca* mRNA levels in sorted TIM4^+^ ResKCs, TIM4^−^ MoKCs, and CD11b^+^ MoMs from the livers, as well as in circulating blood Ly6C^high^ monocytes. Compared to control mice, *Gca* expression was markedly elevated in all three hepatic macrophage subsets in HFFC‐ and HFD‐fed mice (Figure 1G; Figure S1B, Supporting Information). The gating strategy for sorting MoMs (CD45^+^Ly6g^−^CD11b^+^F4/80^−^), ResKC (CD45^+^ Ly6g^−^F4/80^+^TIM4^+^), and MoKC (CD45^+^ Ly6g^−^F4/80^+^TIM4^−^) is shown in Figure S1C (Supporting Information). Immunofluorescence staining further confirmed the increased colocalization of GCA with F4/80^+^ or CD11b^+^ macrophages in MASH mice (Figure 1H; Figure S1D, Supporting Information). Notably, *Gca* expression was also strongly induced in circulating blood Ly6C^high^ monocytes in MASH mice (Figure S1E, Supporting Information), indicating that increased expression of *Gca* in myeloid cells was observed both in the liver and peripheral circulation. Consistent with previous research,^[^ ^17^, ^18^ ^]^ we noticed that during MASH the hepatic macrophage pool primarily consisted of recruited MoMs, whereas ResKCs were diminished (Figure 1I; Figure S1F, Supporting Information), indicating that recruited MoMs were the main contributors to total hepatic *Gca* levels.

To further investigate the clinical relevance of GCA, human liver samples were obtained from 40 and 14 patients with and without MASH, respectively. Patients with MASH expressed higher levels of *GCA* than healthy controls (Figure 1J). Based on the median value of *GCA* detected by quantitative polymerase chain reaction (qPCR), we divided the participants with MASH into two groups: a *GCA* high expression group (20/40, *GCA* ^High^) and a *GCA* low expression group (20/40, *GCA* ^Low^). Hepatic steatosis and macrophage infiltration were more evident in *GCA* ^High^ group than the *GCA* ^Low^ group (Figure 1K). Additionally, Pearson correlation analysis revealed that NAFLD Activity Score (NAS) and triglycerides (TG) were positively correlated with hepatic *GCA* mRNA levels (Figure 1L–M), indicating that the severity of liver disease in patients with MASH was positively correlated with hepatic *GCA* expression. Taken together, these data imply that GCA expression is significantly increased in macrophages of patients and rodents with MASH and correlates with MASH progression.

### 2.2 Effects of GCA on Liver Spheroids

**Figure 2:**
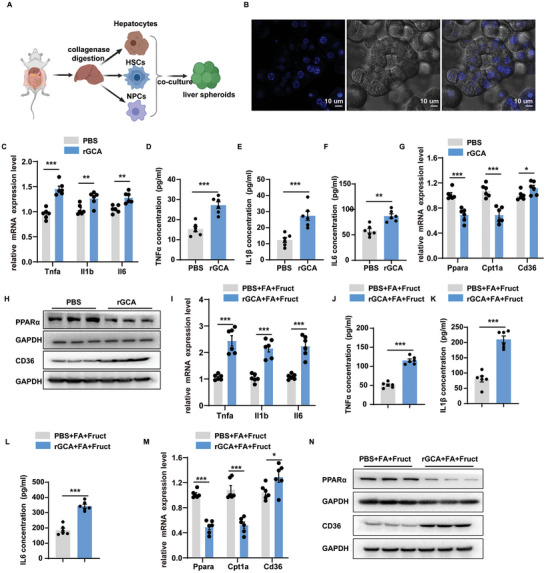
Effects of GCA on liver spheroids. A) Schematic diagram showing primary hepatocytes, non‐parenchymal cells (NPCs), and HSCs were separated from normal mouse livers, and then mixed to constructed a liver spheroid system. This diagram was created with MedPeer.com. B) Images of mouse liver spheroids after 7‐day culture. The nucleuses were stained with Hochest (representative of 3 independent experiments). Scale bar, 10 µm. C) The expression levels of pro‐inflammatory related genes in mouse liver spheroids treated with PBS or rGCA (20 ng mL^−1^) (n = 6 per group). D‐F) The protein secretion of tumor necrosis factor‐α (TNFα), Interleukin‐1β (IL1β), and Interleukin‐ 6 (IL6) in the medium of mouse liver spheroids treated with PBS or rGCA (20 ng mL^−1^) (n = 6 per group). G) The expression levels of fatty acid β‐oxidation related genes (*Ppara* and *Cpt1a*) and fatty acid uptake gens (*Cd36*) in mouse liver spheroids treated with PBS or rGCA (20 ng mL^−1^) (n = 6 per group). H) Western blot analysis of the levels of PPARα and CD36 in mouse liver spheroids treated with PBS or rGCA (n = 3 per group). I) The expression levels of pro‐inflammatory related genes in mouse liver spheroids in the presence of a mixture of fatty acids (FAs) and fructose treated with PBS or rGCA (n = 6 per group). J‐L) The protein secretion of tumor necrosis factor‐α (TNFα), Interleukin‐1β (IL1β), and Interleukin‐ 6 (IL6) in the medium of mouse liver spheroids in the presence of a mixture of fatty acids (FAs) and fructose treated with PBS or rGCA(n = 6). M) The expression levels of fatty acid β‐oxidation related genes (*Ppara* and *Cpt1a*) and fatty acid uptake gens (*Cd36*) in mouse liver spheroids in the presence of a mixture of fatty acids (FAs) and fructose treated with PBS or rGCA(n = 6). N) Western blot analysis of the levels of PPARα and CD36 in mouse liver spheroids in the presence of a mixture of fatty acids (FAs) and fructose treated with PBS or rGCA(n = 3). Data were shown as mean ± SEM. Statistical analysis was assessed by two‐sided Student's t test(C‐G and I‐M). *p < 0.05, **p < 0.01, ***p < 0.001.

According to the previous research,^[^ ^19^ ^]^ primary hepatocytes, NPCs, and HSCs separated from normal mouse livers were mixed to construct a liver spheroid system (**Figure** **2**A). As shown in Figure 2B and Figure S2A (Supporting Information), these cells developed into liver spheroids in 96‐well ultralow adhesion plates after 7‐days of culturing. To assess the effect of GCA in the in vitro culture system, recombinant GCA protein (rGCA) was added to the culture on days 1, 3, and 5 during the culture period and the results were analyzed after 7 days. Compared with phosphate buffered saline (PBS)‐treated group, the liver spheroids treated with rGCA exhibited increased inflammation response (Figure 2C–F), oxidative stress (Figure S2B,C, Supporting Information) and fatty acid uptake, and lower levels of fatty acid β‐oxidation, but no differences in lipogenesis and extent of fibrosis were noted (Figure 2G–H; Figure S2D–G, Supporting Information).

To further approximate the MASH process in vitro, a MASH model was constructed by adding a mixture of fatty acids (FAs) and fructose. Consistent with the basal results, rGCA treatment also remarkably increased inflammation response (Figure 2I–L), oxidative stress (Figure S2H–I, Supporting Information) and fatty acid uptake, and reduced fatty acid β‐oxidation (Figure 2M,N) in MASH models, without impacting on lipogenesis and fibrosis(Figure S2J–M, Supporting Information). Taken together, these results indicate that GCA promotes hepatic proinflammation cytokine secretion and lipid metabolic dysfunction.

### 2.3 rGCA Exacerbates Steatohepatitis

**Figure 3:**
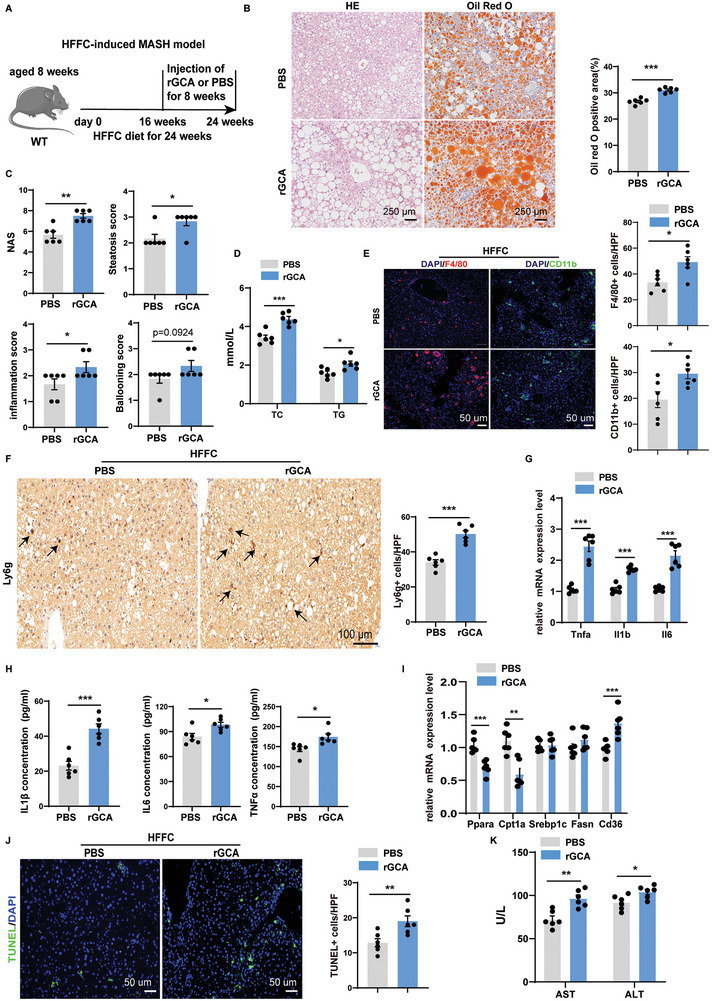
rGCA exacerbates steatohepatitis. A) Schematic diagram showing that 8 weeks old male C57BL/6J mice fed with HFFC diet for 24 weeks were treated with PBS or rGCA. This diagram was created with MedPeer.com. B) Representative images of HE and Oil Red O staining (left) of liver sections in HFFC induced MASH mice treated with PBS or rGCA (n = 6 per group), and quantification of Oil Red O staining (right). The Oil Red O staining positive area was quantified by IPP. Scale bar, 250 µm. C) NAS score was quantified blindly in HFFC induced MASH mice treated with PBS or rGCA (n = 6 per group). D) Serum TG and TC levels in HFFC induced MASH mice treated with PBS or rGCA (n = 6 per group). E) Representative immunofluorescence staining showing the expression of F4/80 (red) and CD11b (green) in the liver of HFFC induced MASH mice treated with PBS or rGCA (n = 6 per group) and quantified as numbers of positive cells per high power field (HPF) (200×). Scale bars: 50 µm. F) Ly6g was detected by immunohistochemistry in the liver of HFFC induced MASH mice treated with PBS or rGCA (n = 6 per group) and quantified as numbers of positive cells per high power field (HPF) (200×). Scale bar, 250 µm. G) Messenger RNA (mRNA) expression of *Tnfa*, *Il‐1b*, and *Il6* was quantified in liver tissues from HFFC induced MASH mice treated with PBS or rGCA (n = 6 per group). H) The concentrations of IL‐1β, IL‐6, and TNF‐α in the serum were measured using ELISA in HFFC induced MASH mice treated with PBS or rGCA (n = 6 per group). I) The expression of fatty acid β‐oxidation related genes, lipogenesis related genes and fatty acid uptake genes in liver tissues from HFFC induced MASH mice treated with PBS or rGCA (n = 6 per group). J) TUNEL (green) staining in the livers of HFFC induced MASH mice treated with PBS or rGCA (n = 6 per group) and quantified as numbers of positive cells per high power field (HPF) (200×). The nucleuses were stained with DAPI. Scale bar, 50 µm. K) Serum AST and ALT levels in HFFC induced MASH mice treated with PBS or rGCA (n = 6 per group). Data were shown as mean ± SEM. Statistical analysis was assessed by two‐sided Student's t test(B‐K). *p < 0.05, **p < 0.01, ***p < 0.001.

After demonstrating that GCA levels are increased in patients and animal models of MASH, and that manipulation of GCA plays a critical role in inflammation and lipid metabolism in liver spheroids in vitro, we next aimed to understand the in vivo functional relevance of our findings. Mice were challenged with an HFFC diet for 24 weeks and rGCA was injected twice per week for 8 weeks via the tail vein (1 mg kg^−1^) (**Figure** **3**A). Compared to PBS‐treated controls, mice administrated with rGCA showed more severe hepatic steatosis in response to HFFC consumption, as evidenced by the results of hematoxylin and eosin ((H&E)) staining and oil red staining (Figure 3B). Consistently, NAS as well as the levels of serum TG and total cholesterol (TC) were also remarkably increased in rGCA‐treated mice (Figure 3C,D). Hepatic inflammation is a key feature of MASH. Mice administrated with rGCA also had greater inflammatory responses than those in control mice as indicated by F4/80, CD11b (macrophage markers), and Ly6g (a neutrophil marker) staining and the mRNA expression levels of proinflammatory genes (Figure 3E–G). Serum IL1β, IL6, and TNFα levels were also elevated in rGCA treated mice (Figure 3H). Moreover, we observed increased expression levels of acid‐uptake‐related genes and decreased expression of beta oxidation genes in rGCA treated mice, although the lipogenesis genes were not significantly different(Figure 3I).Hepatocyte apoptosis was at the center of the transition from hepatic steatosis to MASH. Terminal deoxyribonucleotide transferase‐mediated dUTP nick end labeling (TUNEL) staining showed that hepatocyte apoptosis was increased in rGCA treated mice compared to that in PBS‐treated mice (Figure 3J). Similarly, the serum alanine aminotransferase (ALT) and aspartate aminotransferase (AST), which are the biomarkers of liver injury, were also elevated in mice administrated with rGCA (Figure 3K). However, the liver fibrosis was not significantly different between the two groups as shown by Sirus Red staining and qPCR of fibrosis‐related genes (Figure S3A–B, Supporting Information).

In parallel, the role of rGCA in steatohepatitis was further verified in another MASH mouse model, induced by an MCD diet. Similarly, increased lipid accumulation and hepatic steatosis were observed in rGCA‐treated mice compared to those in control mice (Figure S3C–E, Supporting Information). The results of qPCR showed increased expression levels of acid‐uptake‐related genes and decreased beta‐oxidation genes in rGCA‐treated mice fed with MCD, although the expression of lipogenesis genes was not significantly different(Figure S3F, Supporting Information). Furthermore, heightened inflammation was mirrored by increased infiltration of macrophages and neutrophils, as well as proinflammatory cytokines (Figure S3G–J, Supporting Information). Moreover, hepatocyte apoptosis was intensified in rGCA treated mice as indicated by TUNEL staining (Figure S3K–L, Supporting Information). Consistent with the histological data, the levels of serum biomarkers of liver injury, such as ALT and AST, were also substantially increased in rGCA treated mice (Figure S3M, Supporting Information). In line with the results of HFFC‐induced MASH model, administration of GCA had no obvious effects on hepatic fibrosis (Figure S3N–O, Supporting Information). Taken together, these data suggest that rGCA aggravates hepatic steatosis and inflammation in MASH mice.

### 2.4 *Gca* Deficiency in Myeloid Lineage Ameliorates Steatohepatitis in HFFC‐Induced MASH Models

**Figure 4:**
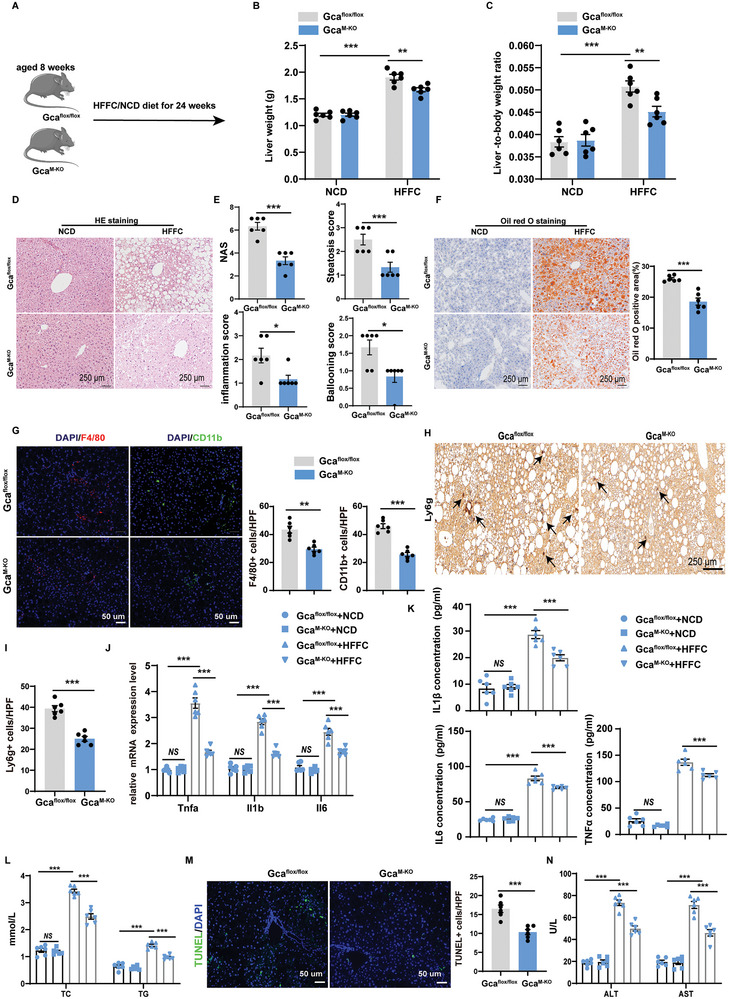
*Gca* deficiency in myeloid lineage ameliorates steatohepatitis in HFFC‐induced MASH models. A) Schematic diagram showing that 8 weeks old male *Gca* ^M‐KO^ or *Gca* ^flox/flox^ mice were fed with HFFC/NCD diet for 24 weeks. This diagram was created with MedPeer.com. B) The liver weight of *Gca* ^M‐KO^ or *Gca* ^flox/flox^ mice fed with HFFC/NCD diet for 24 weeks (n = 6 per group). C) The liver to body weight ratio of *Gca* ^M‐KO^ or *Gca* ^flox/flox^ mice fed with HFFC/NCD diet for 24 weeks (n = 6 per group). D) Representative images of HE staining of liver sections in *Gca* ^M‐KO^ or *Gca* ^flox/flox^ mice fed with HFFC diet or NCD diet for 24 weeks (n = 6 per group). Scale bar, 250 µm. E) NAS score was quantified blindly in *Gca* ^M‐KO^ or *Gca* ^flox/flox^ mice fed with HFFC diet for 24 weeks (n = 6 per group). F) Representative images of Oil Red O staining of liver sections in *Gca* ^M‐KO^ or *Gca* ^flox/flox^ mice fed with HFFC diet or NCD diet for 24 weeks (n = 6 per group), and Oil Red O staining quantification in *Gca* ^M‐KO^ or *Gca* ^flox/flox^ mice fed with HFFC diet for 24 weeks (n = 6 per group). The Oil Red O staining positive area was quantified by IPP. Scale bar, 250 µm. G) Representative immunofluorescence staining showing the expression of F4/80 (red) and CD11b (green) in the liver of *Gca* ^M‐KO^ or *Gca* ^flox/flox^ mice fed with HFFC diet for 24 weeks (n = 6 per group) and quantified as numbers of positive cells per high power field (HPF) (200×). Scale bars: 50 µm. H) Ly6g was detected by immunohistochemistry in the liver of *Gca* ^M‐KO^ or *Gca* ^flox/flox^ mice fed with HFFC diet for 24 weeks (n = 6 per group). Scale bar, 250 µm. I) Quantification of Ly6g^+^ cells in the livers of *Gca* ^M‐KO^ or *Gca* ^flox/flox^ mice fed with HFFC diet for 24 weeks per high power field (HPF) (200×) (n = 6 per group). J) Messenger RNA (mRNA) expression of *Tnfa*, *Il1b* and *Il6* was quantified in liver tissues from *Gca* ^M‐KO^ or *Gca* ^flox/flox^ mice fed with HFFC diet or NCD diet for 24 weeks (n = 6 per group). K) The concentrations of IL‐1β, IL‐6, and TNF‐α in the serum were measured using ELISA in *Gca* ^M‐KO^ or *Gca* ^flox/flox^ mice fed with HFFC diet or NCD diet for 24 weeks (n = 6 per group). L) Serum TC and TG levels in *Gca* ^M‐KO^ or *Gca* ^flox/flox^ mice fed with HFFC diet or NCD diet for 24 weeks (n = 6 per group). M) TUNEL (green) staining in the livers of *Gca* ^M‐KO^ or *Gca* ^flox/flox^ mice fed with HFFC diet for 24 weeks (n = 6 per group), and quantified as numbers of positive cells per high power field (HPF) (200×). The nucleuses were stained with DAPI. Scale bar, 50 µm. N) Serum ALT and AST levels in *Gca* ^M‐KO^ or *Gca* ^flox/flox^ mice fed with HFFC diet or NCD diet for 24 weeks (n = 6 per group). Data were shown as mean ± SEM. Statistical analysis was assessed by two‐sided Student's t test (E‐G, I, and M) or one‐way ANOVA with Tukey's multiple‐comparison test (B‐C, J, K, L, and N). *p < 0.05, **p < 0.01, ***p < 0.001.

To further explore the function of macrophage GCA in liver inflammation, the Cre‐LoxP system was used to create myeloid‐specific *Gca* knockout mice (hereafter referred to as *Gca* ^M‐KO^). Murine MASH models were induced in *Gca* ^flox/flox^ and *Gca* ^M‐KO^ mice fed with HFFC diet for 24 weeks (**Figure** **4**A). As shown in Figure 4B–D, myeloid‐specific *Gca* knockout mice did not have a spontaneous MASH phenotype under normal chow diet condition. Although the body weight and food intake did not differ between *Gca* ^flox/flox^ and *Gca* ^M‐KO^ mice, the liver weight and the liver‐to‐body weight ratio were lower in *Gca* ^M‐KO^ mice fed with HFFC diet (Figure 4B,C; Figure S4A–B, Supporting Information).

*Gca* ^M‐KO^ mice showed improved liver histology with significantly less steatosis and inflammatory cell infiltration than in *Gca* ^flox/flox^ mice fed with HFFC diet (Figure 4D–I). In support of this, the mRNA levels of pro‐inflammatory related genes and acid‐uptake related genes were lower and beta oxidation genes were higher in livers obtained from *Gca* ^M‐KO^ mice fed with HFFC diet than controls (Figure 4J; Figure S4C, Supporting Information). However, the expression level of lipogenesis and fibrosis‐related genes were not significantly different between two groups (Figure S4C–D, Supporting Information). Additionally, serum IL1β, IL6, and TNFα levels were also decreased in *Gca* ^flox/flox^ mice fed with HFFC diet (Figure 4K). Accordingly, the levels of serum TC and TG were significantly decreased in *Gca* ^M‐KO^ mice fed with HFFC diet than in *Gca* ^flox/flox^ mice (Figure 4L). TUNEL staining and serum ALT and AST levels also indicated relief from liver injury (Figure 4M,N). Overall, these data indicate that conditional deletion of *Gca* in myeloid lineage improves HFFC‐induced hepatic steatosis and inflammation.

### 2.5 *Gca* Deficiency in Myeloid Lineage Thwarts Liver Steatosis and Inflammation in HFD‐Induced and MCD‐Induced MASH Models

**Figure 5:**
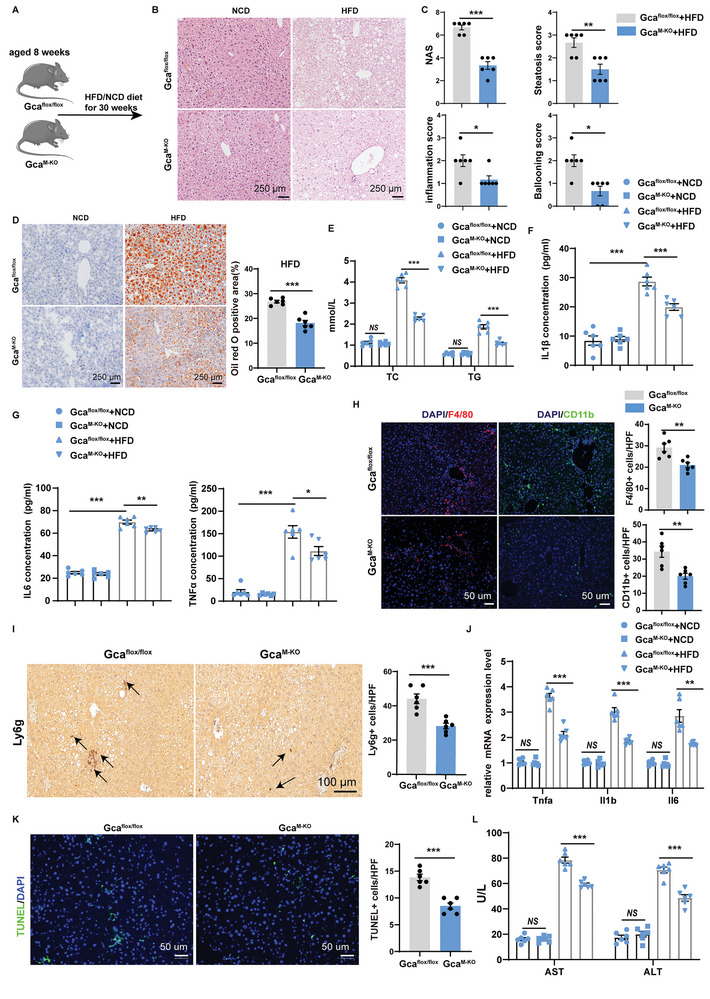
Gca deficiency in myeloid lineage thwarts liver steatosis and inflammation in HFD‐induced MASH models. A) Schematic diagram showing that 8 weeks old male *Gca* ^M‐KO^ or *Gca* ^flox/flox^ mice were fed with HFD/NCD diet for 30 weeks. This diagram was created with MedPeer.com. B) Representative images of HE of liver sections in *Gca* ^M‐KO^ or *Gca* ^flox/flox^ mice fed with HFD diet or NCD diet for 30 weeks (n = 6 per group). Scale bar, 250 µm. C) NAS score was quantified blindly in *Gca* ^M‐KO^ or *Gca* ^flox/flox^ mice fed with HFD diet for 30 weeks (n = 6 per group). D) Representative images of Oil Red O staining of liver sections in *Gca* ^M‐KO^ or *Gca* ^flox/flox^ mice fed with HFD diet or NCD diet for 30 weeks (n = 6 per group), and Oil Red O staining quantification in *Gca* ^M‐KO^ or *Gca* ^flox/flox^ mice fed with HFD diet for 30 weeks (n = 6 per group). The Oil Red O staining positive area was quantified by IPP. Scale bar, 250 µm. E) Serum TC and TG levels in *Gca* ^M‐KO^ or *Gca* ^flox/flox^ mice fed with HFD diet or NCD diet for 30 weeks (n = 6 per group). F) The concentrations of IL‐1β in the serum were measured using ELISA in *Gca* ^M‐KO^ or *Gca* ^flox/flox^ mice fed with HFD diet or NCD diet for 30 weeks (n = 6 per group). G) The concentrations of IL‐6 and TNF‐α in the serum were measured using ELISA in *Gca* ^M‐KO^ or *Gca* ^flox/flox^ mice fed with HFD diet or NCD diet for 30 weeks (n = 6 per group). H) Representative immunofluorescence staining showing the expression of F4/80 (red) and CD11b (green) in the liver of *Gca* ^M‐KO^ or *Gca* ^flox/flox^ mice fed with HFD diet for 30 weeks (n = 6 per group) and quantified as numbers of positive cells per high power field (HPF) (200×). Scale bars: 50 µm. I) Ly6g were detected by immunohistochemistry in the liver of *Gca* ^M‐KO^ or *Gca* ^flox/flox^ mice fed with HFD diet for 30 weeks (n = 6 per group) and quantified as numbers of positive cells per high power field (HPF) (200×). Scale bar, 250 µm. J) Messenger RNA (mRNA) expression of *Tnfa*, *Il1b*, and *Il6* was quantified in liver tissues from *Gca* ^M‐KO^ or *Gca* ^flox/flox^ mice fed with HFD diet or NCD diet for 30 weeks (n = 6 per group). K) TUNEL (green) staining in the liver tissues of *Gca* ^M‐KO^ or *Gca* ^flox/flox^ mice fed with HFD diet for 30 weeks (n = 6 per group), and quantified as numbers of positive cells per high power field (HPF) (200×). The nucleuses were stained with DAPI. Scale bar, 50 µm. L) Serum AST and ALT levels in *Gca* ^M‐KO^ or *Gca* ^flox/flox^ mice fed with HFD diet or NCD diet for 30 weeks (n = 6 per group). Data were shown as mean ± SEM. Statistical analysis was assessed by two‐sided Student's t test(C, D, H‐I and K) or one‐way ANOVA with Tukey's multiple‐comparison test (E‐G, J and L). *p < 0.05, **p < 0.01, ***p < 0.001.

To further confirm the effects of GCA on MASH pathology, we subjected mice to HFD and MCD‐induced MASH (**Figure** **5**A). Similarly, hepatic lipid accumulation was markedly reduced in *Gca* ^M‐KO^ mice as evidenced by hepatic HE, NAS, and Oil Red O staining (Figure 5B–D; Figure S5A–B, Supporting Information). Consistently, the knockout of *Gca* expression mitigated the serum levels of TG and TC, as well as serum IL1β, IL6, and TNFα levels (Figure 5E–G; Figure S5C–D, Supporting Information). We also conducted F4/80, CD11b (macrophage markers), and Ly6g (a neutrophil marker) staining of MASH liver tissues to assess the infiltration of hepatic inflammatory cells. Compared to *Gca* ^flox/flox^ mice, *Gca* ^M‐KO^ mice displayed decreased macrophage and neutrophil accumulation (Figure 5H–I; Figure S5E–F, Supporting Information). In accordance with the staining results, qPCR analysis also detected lower expression level of proinflammation related genes and acid‐uptake related genes and higher expression level of beta oxidation genes in *Gca* ^M‐KO^ mice, although the expression levels of lipogenesis and fibrosis‐related genes were not significantly different between the two groups (Figure 5J; Figure S5G–K, Supporting Information). In addition, we found that hepatocyte apoptosis and serum ALT and AST levels were significantly lower in *Gca* ^M‐KO^ mice (Figure 5K,L; Figure S5L–M, Supporting Information). In conclusion, these findings suggest that *Gca* deficiency in the myeloid lineage thwarts liver steatosis and inflammation in HFD‐induced and MCD‐induced MASH models.

### 2.6 Mechanistic Insights into the Protective Effect of Myeloid *Gca* Depletion on MASH

**Figure 6:**
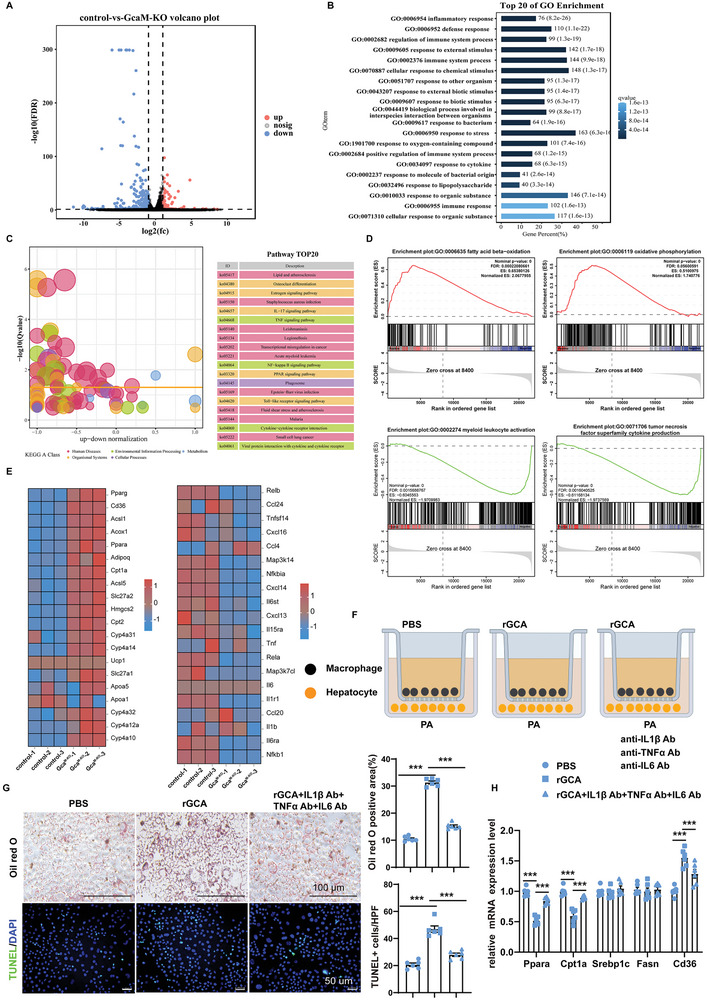
Mechanistic insights into the protective effect of myeloid Gca depletion on MASH. A) Volcano plots showing differentially expressed genes between *Gca* ^flox/flox^ and *Gca* ^M‐KO^ mice fed with HFFC diet for 24 weeks (n = 3 per group). B) GO Biological Process analysis of differentially expressed genes in *Gca* ^flox/flox^ versus *Gca* ^M‐KO^ mice fed with HFFC diet for 24 weeks (n = 3 per group). Top 20 significantly altered pathways are presented. C) KEGG Biological Process analysis of differentially expressed genes in *Gca* ^flox/flox^ versus *Gca* ^M‐KO^ mice fed with HFFC diet for 24 weeks (n = 3 per group). Top 20 significantly altered pathways are presented. D) Gene Set Enrichment Analysis (GSEA) of differentially expressed genes in *Gca* ^flox/flox^ versus *Gca* ^M‐KO^ mice fed with HFFC diet for 24 weeks (n = 3 per group). Significantly altered pathways are presented. E) Heatmap of differentially expressed genes in *Gca* ^flox/flox^ versus *Gca* ^M‐KO^ mice fed with HFFC diet for 24 weeks (n = 3 per group). F) Schematic diagram showing that primary hepatocytes were cocultured with hepatic macrophages and treated with PA, rGCA/PBS and/or appropriate antibodies. This diagram was created with MedPeer.com. G) Effect of rGCA with/without neutralizing antibodies against cytokines treatment on lipogenesis (Oil Red O; scale bar, 100 µm) and apoptosis (TUNEL, scale bar, 50 µm) of hepatocytes cocultured with hepatic macrophages in the presence PA (n = 6 per group). The quantification of Oil Red O staining and TUNEL staining in hepatocytes(right). The Oil Red O staining positive area was quantified by IPP. H) Messenger RNA (mRNA) expression of *Ppara*, *Cpt1a*, *Srebp1c*, *Fasn*, and *Cd36* was quantified in hepatocytes which were cocultured with hepatic macrophages (n = 6 per group). Data were shown as mean ± SEM. Statistical analysis was assessed by one‐way ANOVA with Tukey's multiple‐comparison test (G‐H). *p < 0.05, **p < 0.01, ***p < 0.001.

To further investigate the possible mechanisms underlying the protective effects of myeloid *Gca* depletion on MASH, we assayed the liver tissues of HFFC diet–fed (24 weeks) *Gca* ^M‐KO^ mice and their WT littermates using RNA sequencing (RNA‐Seq), followed by comparative transcriptome analysis (Figure S6A–B, Supporting Information). This unbiased approach identified 466 differentially expressed genes (log_2_(fold change) > 2, FDR(P_adj_) < 0.05), including 136 upregulated and 330 downregulated genes (**Figure** **6**A; Figure S6C, Supporting Information). Gene Ontology (GO) and Kyoto Encyclopedia of Genes and Genomes (KEGG) pathway analyses showed that these differentially expressed genes were implicated in diverse pathways, especially inflammatory responses and lipid metabolism process (Figure 6B–C). Consistently, Gene Set Enrichment Analysis (GSEA) further confirmed the activation of fatty acid oxidation(FAO) related signals, including acyl‐CoA metabolic process, fatty acid beta‐oxidation, oxidative phosphorylation and mitochondrial ATP synthesis coupled electron transport, and the inhibition of inflammation associated signals, such as regulation of tumor necrosis factor superfamily cytokine production, myeloid leukocyte activation, chemokine production and regulation of innate immune response(Figure 6D; Figure S6D, Supporting Information). In addition, the expression levels of fatty acid β‐oxidation related genes were increased and those of inflammatory‐related genes were reduced as shown by a heatmap (Figure 6E). These results are consistent with the improvement in hepatic steatosis and inflammation observed in *Gca* ^M‐KO^ mice.

As hepatocytes are the main cells involved in hepatic steatosis, we then explored the specific influence of GCA on hepatocytes in vitro. Primary hepatocytes were incubated with GCA or PBS during the process of palmitate acid (PA)‐induced lipogenesis. Oil red staining and TUNEL staining showed that GCA had no direct effect on PA‐induced lipogenesis and apoptosis in hepatocytes (Figure S6E–F, Supporting Information). Considering that macrophages‐derived proinflammatory cytokines (IL‐1β, IL‐6, and TNF‐α) may regulate hepatocyte steatosis and apoptosis,^[^ ^20^ ^]^ we constructed a coculture system to observe whether a crosstalk exists between these two cell types (Figure 6F). Primary hepatocytes co‐cultured with GCA‐treated macrophages exhibited increased PA‐induced lipid formation and hepatocyte apoptosis (Figure 6G). Correspondingly, a downregulation in various fatty acid β‐oxidation genes and upregulation of fatty acid uptake gene were also observed in hepatocytes that were cocultured with GCA‐treated macrophages (Figure 6H). However, addition of anti‐IL‐1β, anti‐IL‐6, and anti‐TNF‐α antibodies in the culture medium abolished the functional role of GCA (Figure 6G–H). These results indicate that macrophages are required for the effects of GCA on lipid accumulation and hepatocyte apoptosis.

### 2.7 GCA Favors Hepatic Inflammation via Activating TLR9‐NFκB Signaling in Macrophages

**Figure 7:**
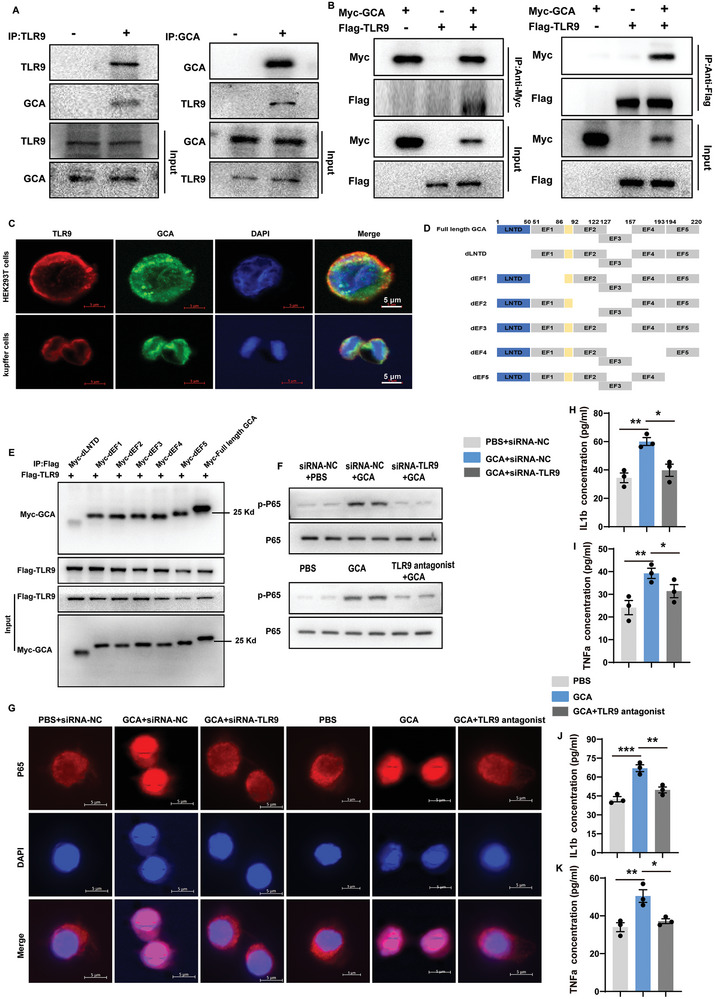
GCA favors hepatic inflammation via activating TLR9‐NFκB signaling in macrophages. A) IP and western blot analysis of the indicated proteins in the primary hepatic macrophages. B) IP and western blot analysis of the indicated proteins in Myc‐GCA and HA‐TLR9 transfected HEK293T cells. C) Representative images of immunofluorescence showed that GCA (green) colocalized with TLR9 (red) in the cytoplasm and membrane of primary hepatic macrophages and Myc‐GCA and HA‐TLR9 transfected HEK293T cells(representative of 3 independent experiments). The nucleuses were stained with DAPI. Scale bar, 5 µm. D) Schematic representation of the functional domains and six deletion mutants of mouse *Gca*. dLNTD, deletion of lipophilic N‐terminal extension domain (LNTD); dEF1‐5, deletion of EF hand motifs 1–5. E) IP analysis of various *Gca* deletion mutants and their binding to full‐length TLR9. F) Representative images of western blotting analysis of p‐P65 in primary hepatic macrophages with addition of 2 µM CpG‐ODN 2088 (TLR9 antagonist) or TLR9 siRNA and with or without GCA treatment. G) Representative immunofluorescent images of P65 (red) in primary hepatic macrophages with addition of PBS+siRNA‐NC, GCA+siRNA‐NC, or GCA+siRNA‐TLR9. The nucleuses were stained with DAPI. Scale bar, 5 µm. H‐I) The protein secretion of IL1β (H) and TNFα(I) in the culture of primary hepatic macrophages with addition of PBS+siRNA‐NC, GCA+siRNA‐NC or GCA+siRNA‐TLR9. J‐K) The protein secretion of IL1β (J) and TNFα(K) in the culture of primary hepatic macrophages with addition of PBS, GCA or GCA+TLR9 antagonist. Data were shown as mean ± SEM. Statistical analysis was assessed by one‐way ANOVA with Tukey's multiple‐comparison test (H‐K). *p < 0.05, **p < 0.01, ***p < 0.001.

Given that inflammatory mechanisms are involved in the entire spectrum of MASLD, macrophages play a pivotal role in propelling MASLD pathogenesis.^[^ ^21^ ^]^ In this study, we explored the effects of GCA on hepatic macrophages. A previous study revealed that Toll‐Like Receptor 9 (TLR9) is a critical driver of MASH pathogenesis,^[^ ^22^ ^]^ and we wondered whether GCA plays a role in MASH via TLR9 signaling. Consistent with the previous study,^[^ ^23^ ^]^ we confirmed the direct binding of GCA with TLR9 via co‐inmunoprecipitation (COIP) analysis. As expected, GCA efficiently coimmunoprecipitated with TLR9 in both macrophages and Myc‐GCA‐ and HA‐TLR9‐transfected HEK293T cells (**Figure** **7**A–B). Transmembrane helix prediction algorithms also predicted that TLR9 is a multiple transmembrane hydrophilic protein, with multiple hydrophobic centers (hydrophobicity score greater than 0) and hydrophilic ends (Figure S7A, Supporting Information). In addition, confocal immunofluorescence analysis revealed that GCA co‐localized with TLR9 in the cytoplasm and membrane of macrophages and Myc‐GCA and HA‐TLR9‐transfected HEK293T cells (Figure 7C).

According to the previously reported structural features, the primary structure of GCA includes a lipophilic N‐terminal extension domain (LNTD) of ≈50 acetylated amino acids and five EF calcium‐binding motifs.^[^ ^13^, ^24^ ^]^ Therefore, we further analyzed which domains are critical for the GCA‐TLR9 interaction. Full length (FL) GCA and six deletion mutants of GCA with an MYC tag were constructed using pCMV‐MYC vector (Figure 7D). Plasmids encoding six truncated fragments of GCA each with an MYC tag and TLR9 with a FLAG tag were transfected into HEK293T cells. Cell lysates were subjected to immunoprecipitation with an anti‐FLAG antibody and immunoblotting with an anti‐FLAG or anti‐MYC antibody. The results of COIP revealed that the deletion of LNTD in GCA abolished its interaction with TLR9, indicating that the LNTD of GCA was responsible for the TLR9‐GCA interaction (Figure 7E).

As TLR9 plays an important role in NF‐κB activation and the results of RNA‐seq also indicated the inhibition of NF‐κB signaling in the livers of *Gca* ^M‐KO^ mice (Figure 6C,E), we measured the phosphorylation of p65 in macrophages. Western blot analysis revealed that rGCA treatment promoted the phosphorylation of P65, whereas, inhibition of TLR9 by siRNA or TLR9 antagonist (5 µM ODN INH‐18 [InvivoGen]) abolished the effects of GCA (Figure 7F). Consistently, nuclear translocalization of P65 induced by GCA was also reversed by TLR9 inhibition (Figure 7G). Moreover, as shown in Figure 7H–K and Figure S7B–E (Supporting Information), administration of GCA increased the levels of IL1β, IL‐6, and TNFα, which have been previously reported to increase lipid accumulation and cell death, causing steatosis and inflammation on hepatocytes.^[^ ^25^ ^]^ However, TLR9 blockage blunted the functional role of GCA(Figure 7H–K; Figure S7B–E, Supporting Information). These results suggest that GCA favors hepatic inflammation via activating TLR9‐NF‐κB signaling in macrophages and that GCA‐induced macrophage activation promotes lipid accumulation and hepatocyte apoptosis.

### 2.8 Anti‐GCA Antibodies Halts the Progression of MASH

**Figure 8:**
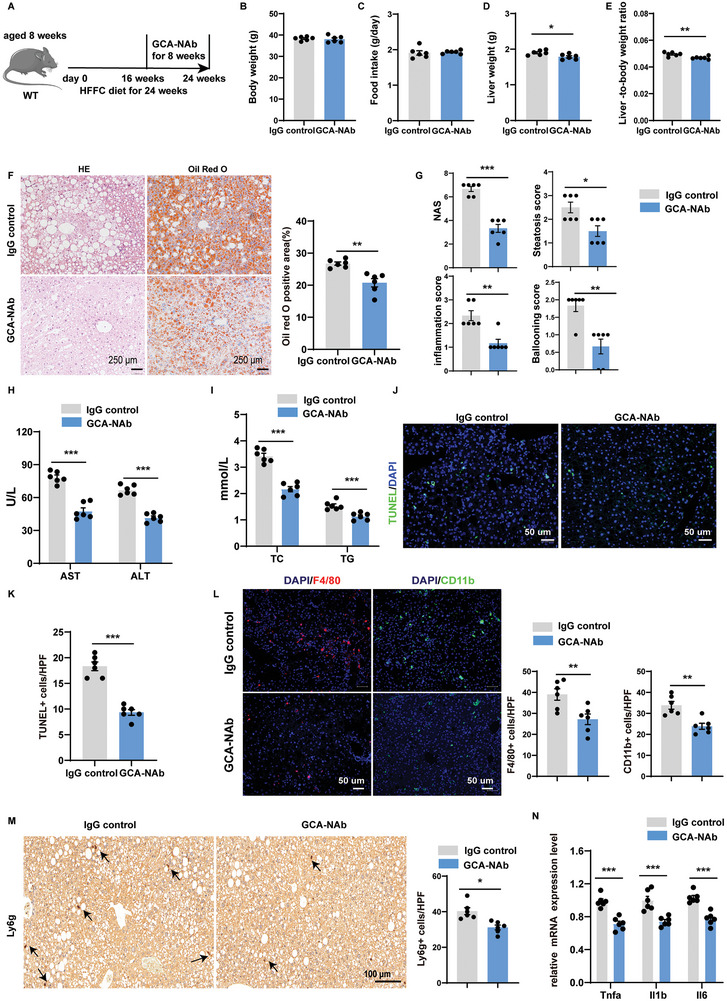
Anti‐GCA antibodies halts the progression of MASH. A) Schematic diagram showing that 8 weeks old male C57BL/6 mice fed with HFFC diet were treated with IgG control or GCA‐NAb. This diagram was created with MedPeer.com. B) The body weight of HFFC diet induced MASH mice treated with IgG or GCA‐NAb (n = 6 per group). C) The daily food intake of HFFC diet induced MASH mice treated with IgG control or GCA‐NAb (n = 6 per group). D) The liver weight of HFFC diet induced MASH mice treated with IgG control or GCA‐NAb (n = 6 per group). E) The liver to body weight ratio of HFFC diet induced MASH mice treated with IgG control or GCA‐NAb (n = 6 per group). F) Representative images of HE and Oil Red O staining of liver sections in HFFC induced MASH mice treated with IgG or GCA‐NAb (n = 6 per group), and Oil Red O staining quantification in HFFC induced MASH mice treated with IgG control or GCA‐NAb (n = 6 per group). The Oil Red O staining positive area was quantified by IPP. Scale bar, 250 µm. G) NAS score was quantified blindly in HFFC induced MASH mice treated with IgG control or GCA‐NAb (n = 6 per group). H) Serum AST and ALT levels in HFFC induced MASH mice treated with IgG control or GCA‐NAb (n = 6 per group). I) Serum TG and TC levels in HFFC induced MASH mice treated with IgG control or GCA‐NAb (n = 6 per group). J) TUNEL (green) staining in the liver of HFFC induced MASH mice treated with IgG or GCA‐NAb (n = 6 per group). The nucleuses were stained with DAPI. Scale bar, 50 µm. K) TUNEL stainings in HFFC induced MASH mice treated with IgG or GCA‐NAb (n = 6 per group) were quantified as numbers of positive cells per high power field (HPF) (200×). L) Representative immunofluorescence staining showing the expression of F4/80(red) and CD11b(green) in the liver of HFFC induced MASH mice treated with IgG control or GCA‐NAb (n = 6 per group)) and quantified as numbers of positive cells per high power field (HPF)(200×). Scale bars: 50 µm. M) Ly6g were detected by immunohistochemistry in the liver of HFFC induced MASH mice treated with IgG control or GCA‐NAb (n = 6 per group), and quantified as numbers of positive cells per high power field (HPF). Scale bar, 250 µm. N) Messenger RNA (mRNA) expression of *Tnfa*, *Il1b* and *Il6* was quantified in liver tissues from HFFC induced MASH mice treated with IgG or GCA‐NAb (n = 6 per group). Data were shown as mean ± SEM. Statistical analysis was assessed by two‐sided Student's t test(B‐I and K‐N). *p < 0.05, **p < 0.01, ***p < 0.001.

Next, we explored the therapeutic potential of targeting GCA in a mouse MASH model induced by 24 weeks of HFFC diet. Mice with HFFC‐induced MASH were intravenously injected with a GCA‐neutralizing antibody (GCA‐NAb, 1 mg/Kg) twice a week for 8 weeks (**Figure** **8**A). Although there was no difference in body weight or food intake, GCA‐neutralizing antibody‐treated mice exhibited lower liver weights and liver weight‐to‐body weight ratios (Figure 8B–E). As depicted by HE and oil red staining, treatment with GCA‐NAb successfully suppressed hepatic lipid accumulation (Figure 8F). Accordingly, NAS and the levels of serum ALT, AST, TG, and TC were significantly decreased in GCA‐NAb treated mice compared to those in control mice (Figure 8G–I). TUNEL staining also showed reduced hepatocyte apoptosis in the GCA‐NAb‐treated mice (Figure 8J,K). Next, the indicators of inflammation in MASH were examined. Consistent with improved hepatic steatosis, less severe inflammation was observed in the GCA‐NAb‐treated group (Figure 8L–N; Figure S8A, Supporting Information). Additionally, the livers of GCA‐NAb treated mice exhibited lower levels of acid‐uptake‐related genes and higher levels of beta‐oxidation genes, although the expression levels of lipogenesis‐ and fibrosis‐related genes were not significantly different between the two groups (Figure S8B–C, Supporting Information).

Then the therapeutic effects of GCA‐NAbs were further validated in MCD‐induced MASH model. Consistently, GCA‐neutralizing antibody ameliorated hepatic lipid accumulation, hepatocyte apoptosis and inflammatory cell infiltration in MCD‐fed mice (Figure S8D–K, Supporting Information). Consistent with the results of HFFC induced NASH, the livers of GCA‐NAb treated mice fed with MCD also displayed lower levels of acid‐uptake related genes and higher levels of beta oxidation genes, although the expression levels of lipogenesis and fibrosis‐related genes were not significantly different between two groups (Figure S8L–M, Supporting Information). Collectively, these data suggest that GCA‐NAb halts the progression of MASH.

## 3 Discussion

MASLD/MASH is a complex, heterogeneous disease. The liver is a dynamic organ that is involved in many physiological and pathological processes. Liver metabolism is regulated by the complex communication and cooperation between hepatocytes, HSCs, macrophages, and other immune cells in the liver,^[^ ^26^ ^]^ providing new insights for MASLD/MASH therapy. However, the interplay between macrophages and hepatocyte metabolism remains largely unknown. In this study, we described a highly significant function of macrophage‐hepatocyte communication in which the upregulation of GCA is involved in the progression of MASH. Our findings greatly extend our current understanding of the pathophysiological role of macrophages in liver metabolism and suggest that GCA may be a potential treatment targets for MASH.

Due to the complexity of the pathophysiology of MASLD/MASH, a single animal disease model cannot fully elucidate its exact pathogenesis. Therefore, to better investigate the role of GCA in the progression of MASLD/MASH, we used multiple classical MASH models. Mice fed an MCD diet rapidly developed hepatic steatohepatitis (≈6–8 weeks), mimicking the impairment observed in patients with MASH. However, it was also accompanied by weight loss, lower insulin levels and lack of insulin resistance.^[^ ^27^ ^]^ MASH developed via HFFC‐induced and HFD‐induced diet is highly similar to that in humans, but it requires a long feeding period and liver fibrosis is often not particularly obvious.^[^ ^28^ ^]^ As expected, the GCA gain/loss‐of‐function enhanced or ameliorated liver steatosis and inflammation in multiple classical MASH models. Furthermore, RNA‐Seq data from the livers of HFFC diet–fed (24 weeks) *Gca* ^M‐KO^ mice and WT littermates identified the activation of FAO‐related signals and the inhibition of inflammation associated signals, which is consistent with the improvement of hepatic steatosis and inflammation in *Gca* ^M‐KO^ mice.

The liver harbors the largest proportion of macrophages among all solid organs in the body, and hepatic macrophages exhibit crucial functions in maintaining liver homeostasis.^[^ ^29^ ^]^ Abnormal macrophage activation has been regarded as a contributing factor to the transition from MASLD to MASH.^[^ ^30^ ^]^ Our results revealed that GCA activates macrophages via TLR9‐NF‐κB signaling, leading to the production of proinflammatory chemokines. Previous studies have reported that TLR9 expression is significantly upregulated in human and murine MASH.^[^ ^22b^ ^]^ The classical pro‐inflammatory role of TLR9 is through the activation of NF‐κB.^[^ ^31^ ^]^ Macrophages produce IL1β, IL6 and TNFα in response to TLR9‐NFκB signaling activation.^[^ ^25^ ^]^ Therefore, we detected the concentration of IL1β and TNFα and observed elevated IL1β and TNFα levels in the medium of GCA treated macrophage. Previous research showed that inflammatory cytokines, including TNFα, IL1β and IL6, promoted inflammation, lipid accumulation and injury in hepatic cells.^[^ ^25^, ^32^ ^]^ Moreover, higher levels of circulating TNFα were also associated with the severity of MASLD.^[^ ^33^ ^]^ Intraperitoneal injection of TNFα evidently promoted the accumulation of hepatic lipid droplet.^[^ ^34^ ^]^ Similarly, we also found that hepatocytes that were cocultured with GCA‐treated macrophages exhibited an augmentation in PA‐induced lipid formation and hepatocyte apoptosis. However, addition of anti‐IL‐1β, anti‐TNF‐αand anti‐IL6 antibodies in the culture medium abolished the functional role of GCA. In contrast, lipotoxicity and hepatocyte apoptosis further attracted and activated macrophages.^[^ ^35^ ^]^

Research and development of therapies for MASLD/MASH have been challenging, as pharmacological agents currently approved for the treatment of MASH are scarce. Until to now, only Rezdiffra, a thyroid hormone receptor beta (THR‐β) agonist, has been approved by the Food and Drug Administration (FDA) for the treatment of MASH (also known as “NASH”). The lack of available drugs can be attributed to various reasons. This is because MASLD/MASH is a complex and heterogeneous disease regulated by multiple intrahepatic and extrahepatic factors. This indicates that MASLD/MASH therapy may require multifunctional effects from drugs or combination therapies. In this study, we developed a GCA‐neutralizing antibody that effectively halted the progression of MASH in multiple murine MASH models. Although there is still a long way to go, this raises the possibility that GCA could emerge as a particularly interesting putative therapeutic target to reverse MASH progression.

Collectively, these findings implicate GCA as a crucial mediator of MASH and reveal a new metabolic signaling axis between hepatocytes and macrophages that provides potential treatment targets for MASH.

## 4 Experimental Section

### Human Liver Samples

This study was approved by the Institutional Ethics Committee of Xiangya Hospital (202103083). All participants signed an informed consent form prior to inclusion in the study. The study protocol was performed in accordance with the current version of the Helsinki Declaration. Human liver samples were obtained from 40 patients with MASH who underwent percutaneous liver biopsy or bariatric surgery at the Xiangya Hospital of Central South University. 14 participants without MASH (patients with hepatic hemangioma) were used as normal controls. All liver specimens were evaluated in a blinded manner by two independent pathologists according to NAS, which is defined as the sum of steatosis, inflammation, and hepatocyte ballooning. Patients with a NAS score ≥ 5 were considered likely to have MASH. Participants with other forms of chronic liver disease or a history of daily alcohol consumption were excluded. The clinical characteristics of the study population are listed in Table S1 (Supporting Information).

### Animal Experiments

The study protocol was approved by the Animal Care and Use Committees of the Laboratory Animal Research Center at the Xiangya Hospital of Central South University (2022020142). All animals were housed under barrier‐specific pathogen‐free (SPF) conditions in individually‐ventilated cages with a 12‐hour dark‐/light‐cycle and ad libitum access to food and water. Only male mice were used for the experiments. Mice were euthanized by carbon dioxide (CO2) asphyxiation inhalation. Cervical dislocation was performed as secondary euthanasia procedure, and tissues were isolated.

To generate genetically modified mice, *Gca* ^flox/flox^(exons 3) mice were obtained from BIORAY LABORATORIES (China), and *Lyz2*‐Cre mice (Stock No: 004781‐B6.129P2‐Lyz2^1(cre)Ifo^/J) were purchased from Jackson Laboratory (USA). Homozygous *Gca* ^flox/flox^ mice were mated with *Lyz2*‐Cre mice to generate myeloid‐specific *Gca* knockout mice (*Gca* ^M‐KO^ mice).

For mice with dietary intervention to induce MASH models, male C57BL/6J mice (8‐12 weeks of age) were supplied with high‐fat/ high‐fructose/ high‐cholesterol (HFFC: protein, 20%; fat, 40%; carbohydrate,40%; cholesterol, 2%; Wuxi Dyets Bioscience Co. Ltd., Wuxi, China) diet for 24 weeks or methionine‐ and choline‐deficient (MCD) diet (Research Diets, A02082002BR, Wuhan BIOPIKE Bioscience Co. Ltd., Wuhan, China) for 8 weeks, or high fat diet (HFD: Rodent Diet With 60 kcal% Fat, Research Diets, Cat#D12492) for 30 weeks. Mice in the control group were fed a normal chow diet (NCD, sws9102, Jiangsu Xietong Pharmaceutical Bio‐engineering Co., Ltd.).

For the administration of rGCA(Novus Biologicals, NBP1‐50967), mice were injected with rGCA twice per week for 8 weeks via tail vein injection (1 mg kg^−1^), and the control mice were treated with equal volumes of PBS. For the treatment of GCA neutralizing antibody (Sino Biological), mice with MASH were intravenously injected with GCA‐neutralizing antibody (GCA‐NAb, 1 mg/Kg) twice a week for eight weeks, and the control mice were treated with equal volumes of IgG control.

### Isolation of Primary Mouse Hepatocytes and Hepatic Macrophages

Primary hepatocytes and hepatic macrophages were isolated as described previously.^[^ ^36^ ^]^ Briefly, 60–80 mL D‐Hanks’ balanced solution was perfused into 6–8 weeks old male mice via inferior vena cava. Then 0.05% IV collagenase (Sigma, Cat#C4‐BIOC) was infused into the liver when the color of the liver changed to beige or light brown. After digestion, the livers were cut into pieces. Cells were detached from the digested liver and suspended in Dulbecco's Modified Eagle Medium (DMEM). The cells suspension was filtered through a 100 µm cell strainer, and then centrifuged at 50 g for 5 min at 4 °C. The resulting pellets contained hepatocytes. Hepatocytes were cultured in DMEM containing 10% fetal bovine serum (Thermo Fisher Scientific). NPCs enriched in the supernatant were centrifuged at 600 g for 6 min, and collected for subsequent use. The Percoll stock solution was diluted to concentrations of 30% and 70%. First, the NPCs were resuspended in 30% Percoll solution. Thereafter, 70% Percoll solution was slowly added to the 30% Percoll solution. The mixture was centrifuged at 1000 g for 30 min. Macrophages appeared as cloud layers between the 30% and the 70% Percoll density layers. These cells were washed twice with PBS and finally cultured in Roswell Park Memorial Institute 1640 (RPMI‐1640) (10% fetal bovine serum and 1% penicillin/streptomycin) at 37 °C.

### Hepatic Stellate Cells (HSC) Isolation

Hepatic stellate cells were isolated as described previously.^[^ ^36a^ ^]^ Briefly, NPCs obtained as described above were centrifuged at 600 g for 6 min. Thereafter 35% Percoll solution, 25% Percoll solution, and the NPC suspension were sequentially added to the centrifuge tube. The cell mixture was centrifuged at 1000 g for 30 min. HSCs appeared between the RPMI‐1640 and 25% Percoll density layers. HSCs were cultured in RPMI‐1640 (10% fetal bovine serum and 1% penicillin/streptomycin) at 37 °C.

### Hepatic Spheroid Culture

The isolation of primary hepatocytes, HSCs and NPCs is described in detail above. Spheroids were cultured in 96‐well ultra‐low adhesion plates. The composition of the spheroid was as follows: cells per well of a 96‐well ultra‐low attachment plate (hepatocyte: HSC: NPC = 1600: 400: 600). An in vitro MASH model was induced by a mixture of fatty acids (FAs) and fructose. Recombinant protein GCA (rGCA, Novus Biologicals, NBP1‐50967) or PBS was added into the spheroids on days 1, 3, and 5 of the culture period. Finally, protein and RNA analyses were performed.

### Plasmid and siRNA Transfection

The mouse *Gca* pcDNA3.1‐MYC‐C plasmid, mouse Tlr9 pcDNA3.1‐FLAG‐C plasmid, mouse *Gca* delete 1–50 pcDNA3.1‐MYC‐C plasmid, mouse *Gca* delete 51–86 pcDNA3.1‐MYC‐C plasmid, mouse *Gca* delete 92–127 pcDNA3.1‐MYC‐C plasmid, mouse *Gca* delete 122–157 pcDNA3.1‐MYC‐C plasmid, mouse *Gca* delete 158–193 pcDNA3.1‐MYC‐C plasmid, mouse *Gca* delete 194–220 pcDNA3.1‐MYC‐C plasmid were constructed by YouBio Technology Corporation (Shanghai, China).

The siRNAs against *Tlr9* and negative control siRNA were synthesized by RiboBio Co. Ltd (Shanghai, China). HEK 293T cells were purchased from Procell Life Science & Technology Co. Ltd. (Wuhan,China) and cultured in DMEM supplemented with 10% fetal bovine serum (Gibco). HEK 293T cell lines were tested for mycoplasma contamination.

For the transfection with siRNA or plasmids, the cells were seeded in 12‐well plates. All transient transfections were conducted using lipofectamine 3000 (Invitrogen) according to the manufacturer's instructions. After 48 h of transfection, the medium was removed and transfection efficiency or functional validation was detected by Western blotting (WB) and qPCR.

### Coculture of Hepatocytes and Hepatic Macrophages

Primary hepatocytes were seeded in a 24‐well Transwell plate (#3450, Corning, USA,) for 24 h, and mouse hepatic macrophages were seeded in the upper chamber. After 1 h, the hepatocytes and macrophages were incubated with 200 µM palmitate (PA) and/or 20 ng mL^−1^ GCA recombinant protein (Recombinant Human Grancalcin Protein, Novus Biologicals, NBP1‐50967) for 24 h at 37 °C. For neutralizing antibody experiments, the concentrations of anti‐TNFα (Thermo Fisher Scientific,Cat#AMC3012), anti‐IL‐1β (Thermo Fisher Scientific,Cat#16‐7012‐81), and anti‐IL‐6 (Thermo Fisher Scientific,Cat#16‐7061‐81) used were 1 µg mL^−1^. After incubation, the cells were subjected to oil red O staining, TUNEL staining and qPCR.

### Cell Oil Red O Staining

Cells were washed twice with PBS and fixed with 4% paraformaldehyde for 30 min. After washing twice with PBS, the cells were stained with freshly diluted Oil Red O working solution (Sigma‐Aldrich, Cat#O0625) for 30 min. Finally, the dish was washed with PBS and the cells were observed under a light microscope.

### Terminal Deoxyribonucleotide Transferase (Tdt)‐Mediated Dutp Nick End Labeling (TUNEL) assay

TUNEL in situ detection of DNA fragments was performed using a one‐step TUNEL apoptosis assay kit (Elabscience,Cat#E‐CK‐A320) according to the manufacturer's instructions.

### Hematoxylin‐Eosin (H&E) Staining and Oil Red O Staining

Livers were fixed with a 4% paraformaldehyde solution and embedded in paraffin wax. Sections were cut at 5 µm using a microtome, and deparaffinized tissue sections were subjected to Oil Red O staining and H&E staining for histological examination. Sliced sections with a thickness of 5 µm were stained with eosin for 3 minutes and hematoxylin for 25 seconds, and then observed under a light microscope.

For Oil Red O staining, slices were removed from the −20 degree freezer and rewarmed at room temperature for 10 min. Frozen sections were stained with oil red O for 20 min. After washing with PBS, the sections were stained with hematoxylin for 20 s, and observed under a light microscope.

### Immunohistochemistry

Paraffin‐embedded slices were subjected to dewaxing, hydration and antigen retrieval. The slices were then washed in PBS, and endogenous peroxidase activity was blocked with 3% H_2_O_2_ for 20 min at room temperature. After washing with PBS three times, the slices were permeabilized with 0.3% Triton X‐100 for 20 min, and were blocked with normal goat serum for 60 min at 37 °C, followed by incubation with primary antibodies (Ly6g, CST, 87048T, 1: 200) against the ly6g antigen overnight at 4 °C. After washed with PBS, the sections were then incubated with secondary antibody for 60 min at 37 °C. The slices were then washed with PBS and incubated for 4 min with diaminobenzidine. After washing with PBS, nuclei were counterstained with hematoxylin. The resulting slices were examined under a light microscope at the photo documentation facility. For each tissue section, the number of positively stained cells was calculated from 6 different 200 × magnification fields under a light microscope.

### Immunofluorescence Staining

Immunofluorescence staining was performed according to standard procedures. Paraffin sections of tissue or cell climbing sheets were incubated with specific primary antibodies (GCA, PA5‐77127, Invitrogen,1:200; F4/80, ab6640, abcam,1:200; TLR9, sc‐52966, Santa Cruz,1: 200; CD11b, abcam, ab52478, 1: 200; CD68, Santa Cruz, sc‐17832, 1: 200) at 4 °C overnight. After washing three times with PBS, the sections or sheets were incubated with the corresponding fluorescent secondary antibodies. Cell nuclei were labeled with DAPI. The cells were imaged using fluorescence microscope or confocal microscope.

### Immunoprecipitation and Western Blot Analysis

Immunoprecipitation was performed as previously described.^[^ ^37^ ^]^ Briefly, cells were lysed in RIPA lysis buffer on ice for 20 min. The total cell lysates were collected and incubated with corresponding antibodies (FLAG, 14793S, Cell Signaling Technology, 1: 50; MYC, 2276S, Cell Signaling Technology, 1: 250; GCA, PA5‐77127, Invitrogen, 1: 100; TLR9, sc‐52966, Santa Cruz, 1: 100) and protein A/G beads at 4 °C overnight. The immunoprecipitates were separated by sodium dodecyl‐sulfate polyacrylamide gel electrophoresis (SDS‐PAGE) and visualized using ECL Plus.

Western blotting was performed as previously described.^[^ ^38^ ^]^ Briefly, tissue or cell lysates were separated by SDS‐PAGE and blotted on PVDF (polyvinylidene difluoride) membranes (Millipore). Thereafter, the lysates were incubated with the corresponding primary antibody (MYC, 2276S, Cell Signaling Technology, 1: 1000; Flag, 14793S, Cell Signaling Technology, 1: 1000; GCA, PA5‐77127, Invitrogen, 1: 1000; TLR9, sc‐52966, Santa Cruz, 1: 1000; SREBF1, 14088‐1‐AP, proteintech, 1: 1000; PPAR alpha, ab24509, Abcam, 1: 1000; CD36, 74 002, Cell Signaling Technology, 1: 1000; COL1A1, 72 026, Cell Signaling Technology, 1: 1000) at 4 °C overnight. Specific proteins were visualized using ECL Plus.

### ELISA

The concentrations of IL‐1β, IL‐6, and TNF‐α in the medium/serum were measured using ELISA kits from Thermo Scientific (Mouse IL‐1 beta ELISA Kit, BMS6002; Mouse IL‐6 Uncoated ELISA, 88‐7064;Mouse TNF alpha Uncoated ELISA, 88–7324).

### Biochemical Analyses

Serum ALT, AST, TG and TC levels were measured according to manufacturer's instructions using commercial kits provided by Elabscience Biotechnology (E‐BC‐K235‐M, E‐BC‐K236‐M, E‐BC‐K261‐M and E‐BC‐K109‐S).

### Detection of Oxidative Stress Related Indicators

The level of superoxide dismutase (SOD) and malondialdehyde (MDA) in liver spheroids were determined by SOD assay kit (A001‐3, Beijing Solarbio Science & Technology Co., Ltd) and MDA assay kit (BC0025, Beijing Solarbio Science & Technology Co., Ltd).

### RNA Sequencing and Analysis

Livers were isolated from the HFFC diet–fed (24 weeks) *Gca* ^M‐KO^ mice and WT littermates. Total RNA was extracted using a TRIzol reagent kit (Invitrogen, Carlsbad, CA, USA) according to the manufacturer's protocol. The RNA quality was assessed on an Agilent 2100 Bioanalyzer (Agilent Technologies, Palo Alto, CA, USA) and checked using RNase‐free agarose gel electrophoresis. After total RNA was extracted, eukaryotic mRNA was enriched by Oligo(dT) bead. Then the enriched mRNA was fragmented into short fragments using fragmentation buffer and reverse transcribed into cDNA by using NEBNext Ultra RNA Library Prep Kit for Illumina (NEB #7530, New England Biolabs, Ipswich, MA, USA). The purified double‐stranded cDNA fragments were end repaired, A base added, and ligated to Illumina sequencing adapters. The ligation reaction was purified with the AMPure XP Beads (1.0X). And polymerase chain reaction (PCR) amplified. The resulting cDNA library was sequenced using Illumina Novaseq6000 by Gene Denovo Biotechnology Co. (Guangzhou, China). The bioinformatics analysis was assisted by Genedenovo Biotechnology Co., Ltd (Guangzhou, China).

### Flow Cytometry and Cell Sorting

The cells were incubated with BV510‐Zombie dye for 15 min at room temperature. Thereafter, the cells were washed with PBS, and incubated with Fixation/Permeabilization solution (BD Biosciences,Cat#554 714) at 4 °C for 30 min. After washing with 1 × BD Perm/Wash buffer(BD Biosciences,Cat#554 714) and centrifuging at 1000 g at 25 °C for 5 min, cells were subsequently stained with fluorochrome‐conjugated antibodies: APC‐Cy7‐CD45, PE‐Cy7‐ly6C, PE‐F4/80, APC‐TIMP4 and FITC‐CD11b antibodies (APC‐Cy7‐CD45, Biolegend, 109 824; PE‐Cy7‐ly6C, Biolegend, 128 018; PE‐F4/80, Biolegend, 123 110; APC‐TIMP4, Biolegend, 130 022; FITC‐CD11b, Biolegend, 101 206) at 4 °C for 45 min. After washing with 1 × BD Perm/Wash buffer (BD Biosciences, Cat#554 714), the cells were resuspended in PBS. All antibodies were diluted according to the manufacturer’ s instructions. Stained cells were collected using BD FACSCanto II system and analyzed using FlowJo V10.

For cell sorting, single‐cell suspension was obtained in the cell platform.The cells were incubated with BV510‐ Zombie Dyes for 15 mins at room temperature. Thereafter, the cells were washed with PBS, and subsequently stained with fluorochrome‐conjugated antibodies (PE‐Cy7‐Ly6C antibody, Biolegend, 128018; APC‐Cy7‐CD45, Biolegend, 109824; PE‐Cy7‐ly6C, Biolegend, 128018; PE‐F4/80, Biolegend, 123110; APC‐TIMP4, Biolegend, 130022; FITC‐CD11b, Biolegend, 101206) at 4 °C for 45 min. Finally, the cells were washed with PBS again and resuspended in PBS. Stained cells were collected using the BD system, and finally the sorted cells were used for mRNA analysis.

### Quantitative Polymerase Chain Reaction(qPCR) Analysis

For qPCR analysis, the total RNAs from cultured cells or tissue were isolated by RNAex Pro RNA reagent (Accurate Biotechnology (Hunan) Co., Ltd) and performed on the Applied Biosystems Quant Studio3. Primer sequences are listed in Table S2 (Supporting Information).

### Statistical Analysis

Data are presented as means ± SEM. Comparisons between two groups were performed with a 2‐tailed unpaired t‐test, and the comparison for 3 or more groups was performed with one‐way ANOVA followed by Tukey's multiple comparison test. To analyze the correlation between GCA with NAS and triglyceride, two‐sided Pearson's correlation test was applied. Statistical significance was calculated and indicated (*p < 0.05, **p < 0.01, and ***p < 0.001). Data analyses were carried out by GraphPad Prism 8.0.

## Conflict of Interest

The authors declare no conflict of interest.

## Author Contributions

T.S. and Y.H. contributed equally to this work. X.L., J.W., and T.S. supervised the project, wrote the manuscript, and obtained funding. Y.H. and T.S. conducted most of the experiments. M.W., H.Z., Y.H., M.Y., Q.G., Y.X., G.C., and M.Z. participated in research design and data discussion. All authors performed data analysis and interpretation, and read and approved the final manuscript.

## Supporting information



Supporting Information

## Data Availability

The data that support the findings of this study are available from the corresponding author upon reasonable request.
