# Supplementary material for: Macrophage‐Hepatocyte Circuits Mediated by Grancalcin Aggravate the Progression of Metabolic Dysfunction Associated Steatohepatitis
Source: Adv Sci (Weinh). 2024 Sep 16;11(42):2406500. doi: 10.1002/advs.202406500 (PMC11558151; doi:10.1002/advs.202406500)
Supplement: Supplementary file 1 — Supporting Information [file ADVS-11-2406500-s001.docx]

Supplementary Materials for

**Macrophage-hepatocyte circuits mediated by grancalcin aggravate the progression of metabolic dysfunction associated steatohepatitis**

Tian Su ^1,#^ ,Yue He^1,#^, Min Wang^1^, Haiyan Zhou^1^, Yan Huang^1^, Mingsheng Ye^1^, Qi Guo^1^, Ye Xiao^1^, Guangping Cai^1^, Mingyang Zhao^1^, Jianping Wang^2*^, Xianghang Luo^1，3*^

^#^ These authors contributed equally: Tian Su, Yue He.

* Corresponding authors

**This PDF file includes:**

1. Supplementary Figures and Figure Legends S1-S8
2. Supplementary Tables S1-S2

**Supplementary Figures**

**
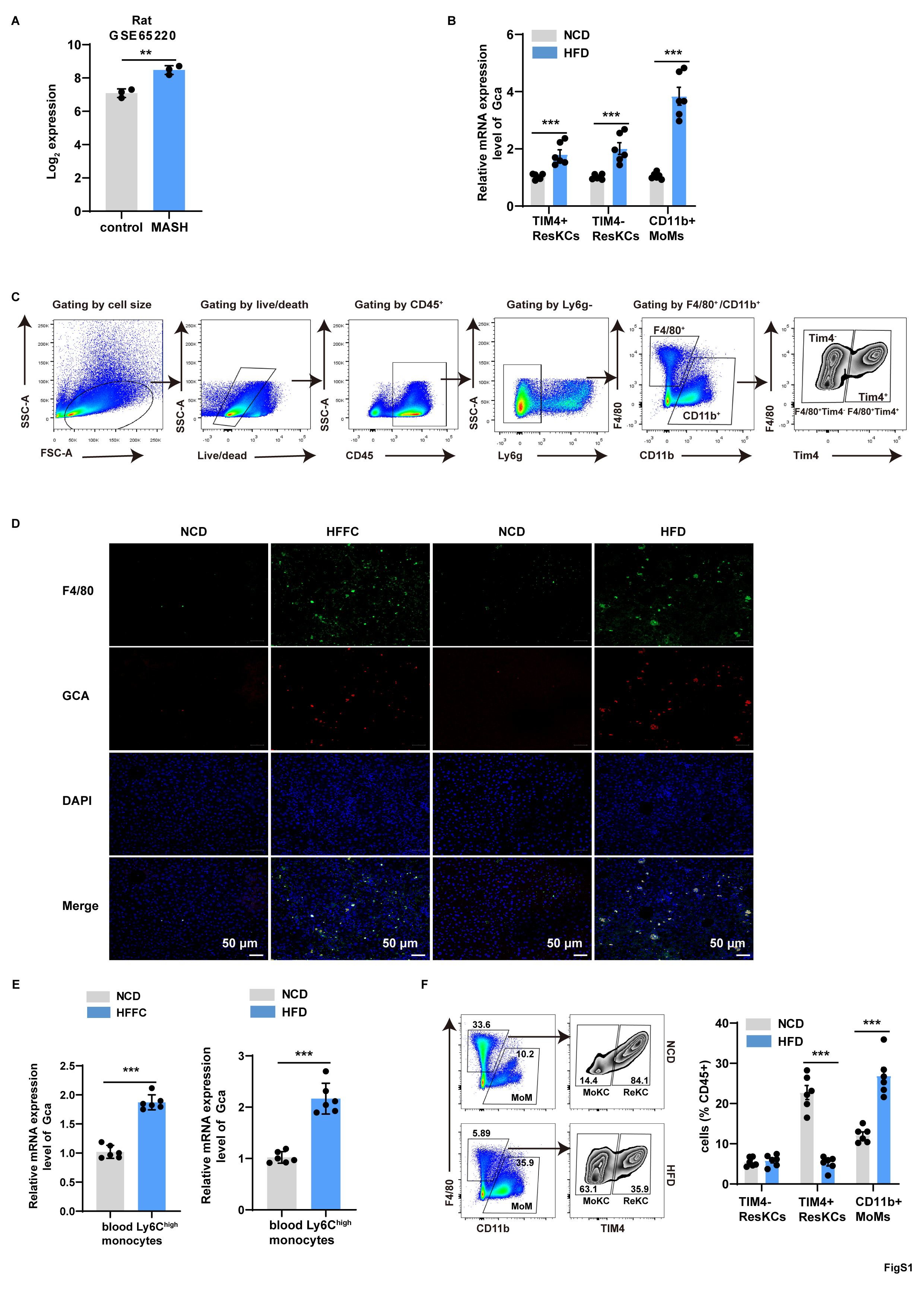
Figure S1. The expression of GCA is upregulated in macrophages from patients and rodents with MASH and correlates with MASH progression.**

1. Liver *Gca* expression among rats with metabolic dysfunction-associated steatohepatitis. (MASH) or healthy controls as determined by microarray data(n=3).
2. Expression of *Gca* in hepatic CD45^+^Ly6g^-^F4/80^+^TIM4^+^ ResKC, CD45^+^Ly6g^-^F4/80^+^TIM4^-^ MoKC and CD45^+^Ly6g^-^F4/80^-^CD11b^+^ MoMs sorted from the livers of mice fed with normal chow diet (NCD) or high fat diet (HFD) diet (n=6 per group).
3. Representative images showing gating strategy for sorting of CD45+Ly6g^-^F4/80^+^TIM4^+^ ResKC, CD45^+^Ly6g^-^F4/80^+^TIM4- MoKC and CD45^+^Ly6g^-^F4/80^-^CD11b^+^ MoMs from the livers of control mice or MASH mice.
4. Representative immunofluorescence staining showing the expression of GCA (red) and F4/80 (green) in the livers of mice fed NCD or HFD diet (n =6 per group). Scale bars: 50 μm..
5. Expression of *Gca* in Ly6C^high^ monocytes sorted from the blood of mice fed with normal chow diet (NCD) or HFFC/HFD diet (n=6 per group).
6. Flow cytometry analysis of CD45^+^Ly6g^-^F4/80^+^TIM4^+^ ResKC, CD45^+^Ly6g^-^F4/80^+^TIM4^-^ MoKC and CD45^+^Ly6g^-^F4/80^-^CD11b^+^ MoMs in the livers of mice fed NCD or HFD diet (n = 6 per group).

Data were shown as mean ± SEM. Statistical analysis was assessed by two-sided Student’s t test (A-B,E-F). *p<0.05, **p<0.01, ***p<0.001.


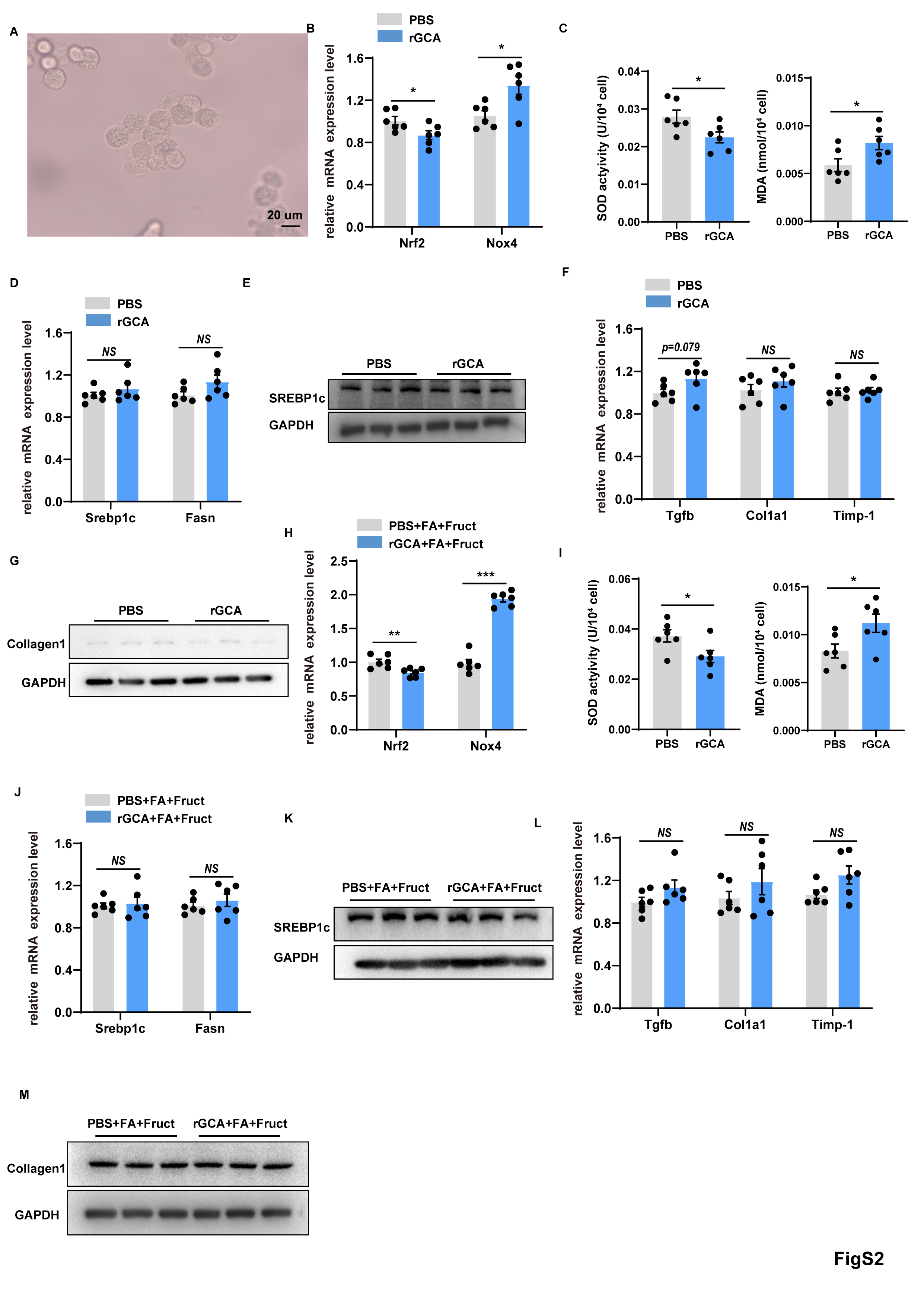


**Figure S2. Effects of GCA on liver spheroids**

1. Images of mouse liver spheroids after 7-day culture under white light. Scale bar, 20 μm.
2. QPCR analysis of the expression levels of *Nrf2* and *Nox4* in mouse liver spheroids treated with PBS or rGCA (n = 6 per group).
3. Superoxide dismutase (SOD) activity and Malondialdehyde (MDA) level in mouse liver spheroids treated with PBS or rGCA (n = 6 per group).
4. QPCR analysis of the expression levels of lipogenesis related genes (*Srebp1c* and *Fasn)* in mouse liver spheroids treated with PBS or rGCA (n = 6 per group).
5. Western blot analysis of the levels of SREBP1c in mouse liver spheroids treated with PBS or rGCA (n = 3 per group).
6. QPCR analysis of the expression levels of fibrosis related genes *(Tgfb*, *Col1a1* and *Timp1*) in mouse liver spheroids treated with PBS or rGCA (n = 6 per group).
7. Western blot analysis of the levels of Collagen1 in mouse liver spheroids treated with PBS or rGCA (n = 3 per group).
8. QPCR analysis of the expression levels of *Nrf2* and *Nox4* in mouse liver spheroids in the presence of a mixture of fatty acids (FAs) and fructose treated with PBS or rGCA(n=6).
9. Superoxide dismutase (SOD) activity and Malondialdehyde (MDA) level in mouse liver spheroids in the presence of a mixture of fatty acids (FAs) and fructose treated with PBS or rGCA(n=6).
10. QPCR analysis of the expression levels of lipogenesis related genes (*Srebp1c* and *Fasn)* in mouse liver spheroids in the presence of a mixture of fatty acids (FAs) and fructose treated with PBS or rGCA(n=6).
11. Western blot analysis of the levels of SREBP1c in mouse liver spheroids in the presence of a mixture of fatty acids (FAs) and fructose treated with PBS or rGCA(n=3).
12. QPCR analysis of the expression levels of fibrosis related genes *(Tgfb*, *Col1a1* and *Timp1*) in mouse liver spheroids in the presence of a mixture of fatty acids (FAs) and fructose treated with PBS or rGCA(n=6).
13. Western blot analysis of the levels of Collagen1 in mouse liver spheroids in mouse liver spheroids in the presence of a mixture of fatty acids (FAs) and fructose treated with PBS or rGCA(n=3).

Data were shown as mean ± SEM. Statistical analysis was assessed by two-sided Student’s t test(B-D, F, H-J and L). *p<0.05, **p<0.01, ***p<0.001.


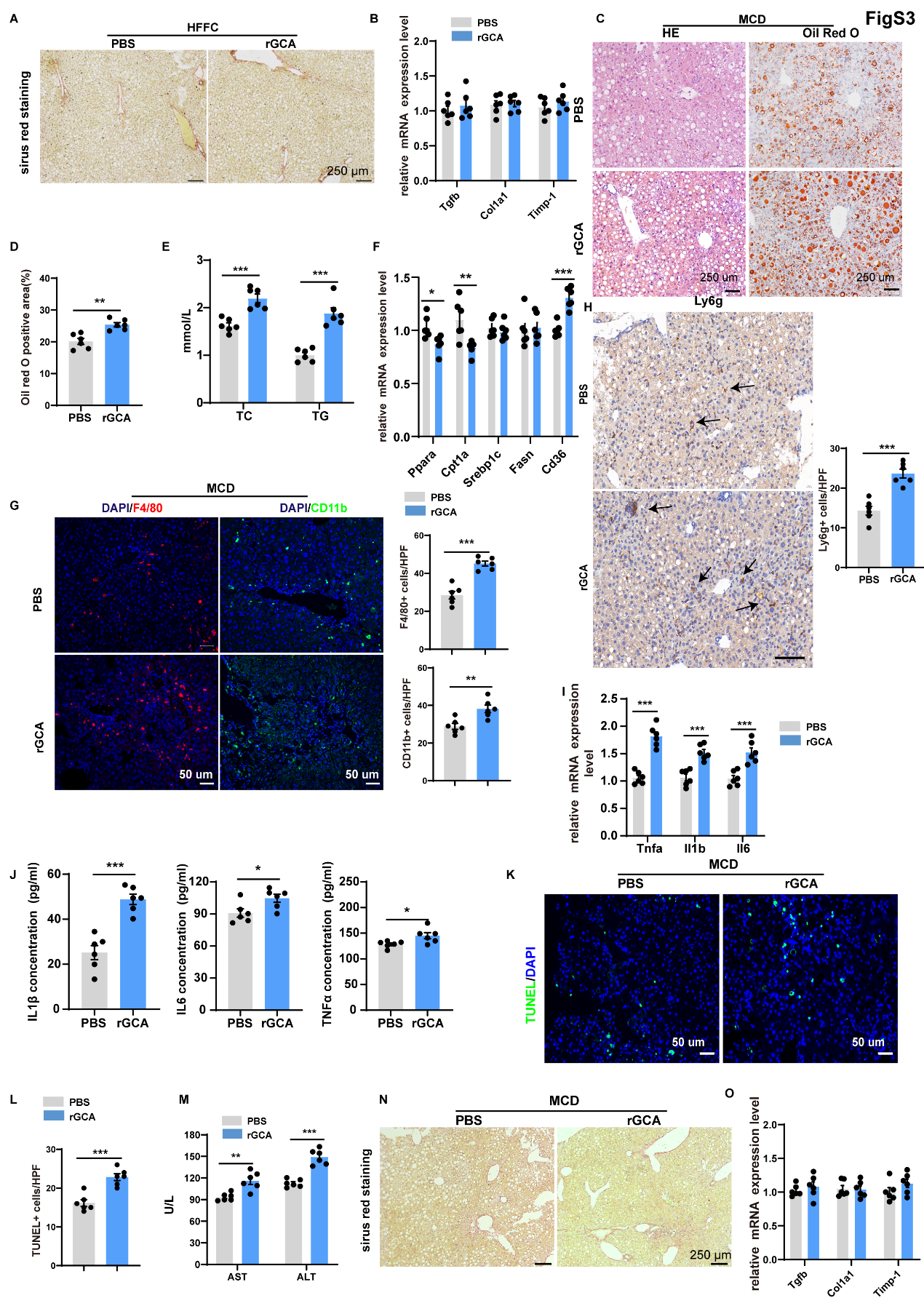


**Figure S3. rGCA exacerbates steatohepatitis**

1. The degree of fibrosis was evaluated by Sirius Red staining in HFFC induced MASH mice treated with PBS or rGCA (n=6 per group). Scale bar, 250 μm.
2. The expression of fibrosis related genes in liver tissues from HFFC induced MASH mice treated with PBS or rGCA (n=6 per group).
3. Representative images of HE and Oil Red O staining of liver sections in MCD induced MASH mice treated with PBS or rGCA (n=6 per group). Scale bar, 250 μm.
4. Quantification of Oil Red O staining. The Oil Red O staining positive area was quantified by IPP.
5. Serum TG and TC levels in MCD induced MASH mice treated with PBS or rGCA (n=6 per group).
6. The expression of fatty acid β-oxidation related genes, lipogenesis related genes and fatty acid uptake genes in liver tissues from MCD induced MASH mice treated with PBS or rGCA (n=6 per group).
7. Representative immunofluorescence staining showing the expression of F4/80 (red) and CD11b (green) in the liver of MCD induced MASH mice treated with PBS or rGCA (n=6 per group) and quantified as numbers of positive cells per high power field (HPF) (200×). Scale bars: 50 μm.
8. Ly6g were detected by immunohistochemistry in the liver of MCD induced MASH mice treated with PBS or rGCA (n=6 per group) and quantified as numbers of positive cells per high power field (HPF) (200×). Scale bar, 250 μm.
9. Messenger RNA (mRNA) expression of *Tnfa*, *Il1b* and *Il6* was quantified in liver tissues from MCD induced MASH mice treated with PBS or rGCA (n=6 per group).
10. The concentrations of IL-1β，IL-6 and TNF-α in the serum were measured using ELISA in MCD induced MASH mice treated with PBS or rGCA (n=6 per group).

(K-L) TUNEL (green) staining in the livers of MCD induced MASH mice treated with PBS or rGCA (n=6 per group) and quantified as numbers of positive cells per high power field (HPF) (200×). The nucleuses were stained with DAPI. Scale bar, 50 μm.

1. Serum AST and ALT levels in MCD induced MASH mice treated with PBS or rGCA (n=6 per group).
2. The degree of fibrosis was evaluated by Sirius Red staining in MCD induced MASH mice treated with PBS or rGCA (n=6 per group). Scale bar, 250 μm.
3. The expression of fibrosis related genes in liver tissues from MCD induced MASH mice treated with PBS or rGCA (n=6 per group).

Data were shown as mean ± SEM. Statistical analysis was assessed by two-sided Student’s t test(B, D-J, L-M and O). **p*<0.05, ***p*<0.01, ****p*<0.001.


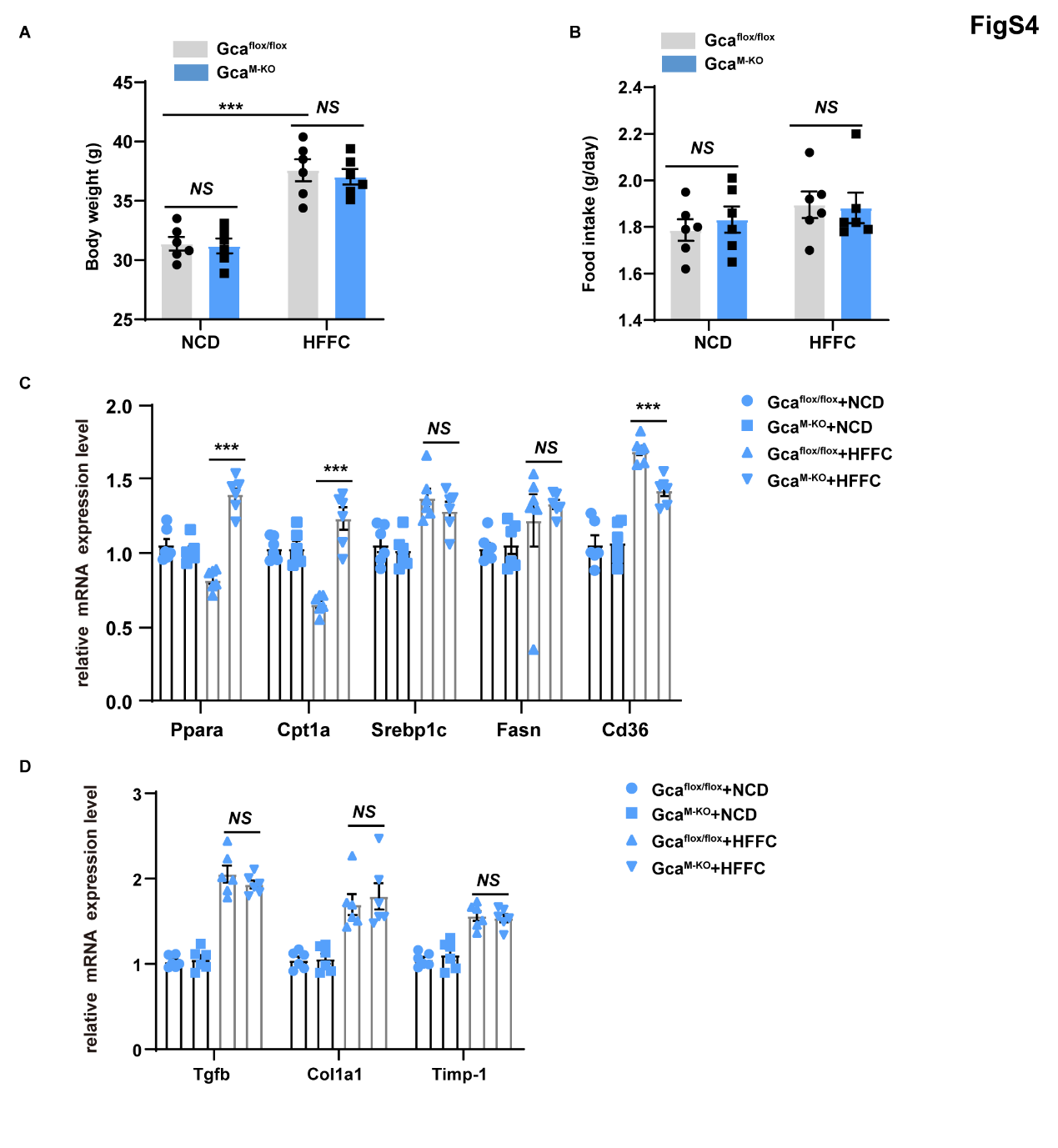


**Fig. S4. *Gca* deficiency in myeloid lineage ameliorates steatohepatitis in HFFC-induced MASH models**

1. The body weight of *Gca*^M-KO^ or *Gca*^flox/flox^ mice fed with HFFC diet or NCD diet for 24 weeks (n=6 per group).
2. The daily food intake of *Gca*^M-KO^ or *Gca*^flox/flox^ mice fed with HFFC die NCD diet for 24 weeks (n=6 per group).
3. Relative mRNA levels of genes related to fatty acid metabolism in the livers of *Gca*^M-KO^ or *Gca*^flox/flox^ mice fed with HFFC diet or NCD diet for 24 weeks (n=6 per group).
4. The expression of fibrosis related genes in liver tissues of *Gca*^M-KO^ or *Gca*^flox/flox^ mice fed with HFFC diet or NCD diet for 24 weeks (n=6 per group).

Data were shown as mean ± SEM. Statistical analysis was assessed by one-way ANOVA with Tukey’s multiple-comparison test (A-D). *p<0.05, **p<0.01, ***p<0.001.


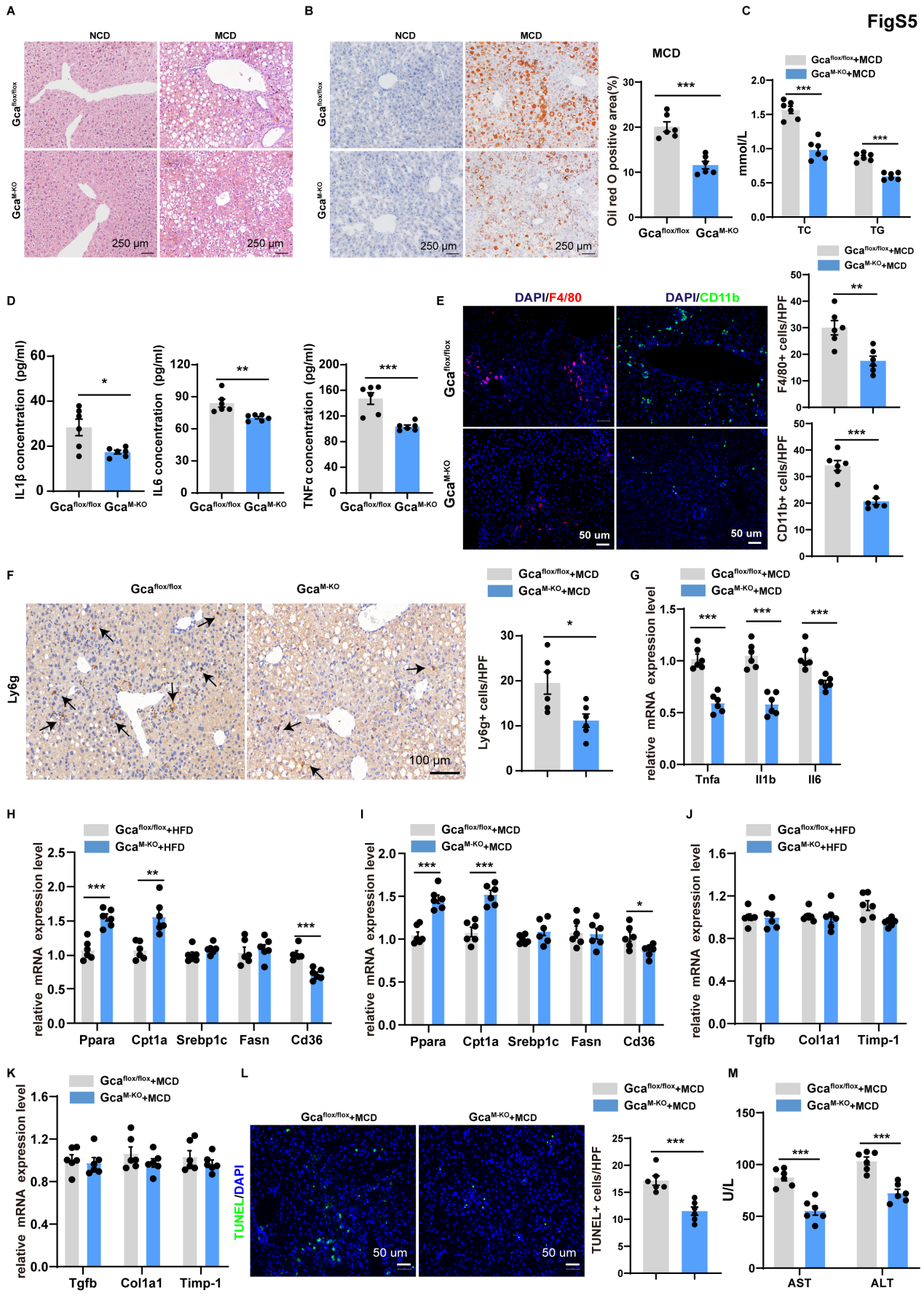


**Figure S5. *Gca* deficiency in myeloid lineage thwarts liver steatosis and inflammation in MCD-induced MASH models**

1. Representative images of HE of liver sections in *Gca*^M-KO^ or *Gca*^flox/flox^ mice fed with MCD diet or NCD diet for 8 weeks (n=6 per group). Scale bar, 250 μm.
2. Representative images of Oil Red O staining of liver sections and Oil Red O staining quantification in *Gca*^M-KO^ or *Gca*^flox/flox^ mice fed with MCD diet or NCD diet for 8 weeks (n=6 per group). The Oil Red O staining positive area was quantified by IPP. Scale bar, 250 μm.
3. Serum TC and TG levels in *Gca*^M-KO^ or *Gca*^flox/flox^ mice fed with MCD diet or NCD diet for 8 weeks (n=6 per group).
4. The concentrations of IL-1β，IL-6 and TNF-α in the serum were measured using ELISA in *Gca*^M-KO^ or *Gca*^flox/flox^ mice fed with MCD diet or NCD diet for 8 weeks (n=6 per group).
5. Representative immunofluorescence staining showing the expression of F4/80 (red) and CD11b (green) in the liver of *Gca*^M-KO^ or *Gca*^flox/flox^ mice fed with MCD diet for 8 weeks (n=6 per group), and immunofluorescence staining were quantified as numbers of positive cells per high power field (HPF) (200×) (right). Scale bar, 50 μm.
6. Ly6g were detected by immunohistochemistry in the liver of *Gca*^M-KO^ or *Gca*^flox/flox^ mice fed with MCD diet for 8 weeks (n=6 per group) and quantified as numbers of positive cells per high power field (HPF) (200×). Scale bar, 250 μm.
7. Messenger RNA (mRNA) expression of *Tnfa*, *Il1b* and *Il6* was quantified in liver tissues from *Gca*^M-KO^ or *Gca*^flox/flox^ mice fed with MCD diet or NCD diet for 8 weeks (n=6 per group).
8. Relative mRNA levels of genes related to fatty acid metabolism in the livers of *Gca*^M-KO^ or *Gca*^flox/flox^ mice fed with HFD diet or NCD diet for 30 weeks (n=6 per group).
9. Relative mRNA levels of genes related to fatty acid metabolism in the livers of *Gca*^M-KO^ or *Gca*^flox/flox^ mice fed with MCD diet or NCD diet for 8 weeks (n=6 per group).
10. The expression of fibrosis related genes in liver tissues of *Gca*^M-KO^ or *Gca*^flox/flox^ mice fed with HFD diet or NCD diet for 30 weeks (n=6 per group).
11. The expression of fibrosis related genes in liver tissues of *Gca*^M-KO^ or *Gca*^flox/flox^ mice fed with MCD diet or NCD diet for 8 weeks (n=6 per group).
12. TUNEL (green) staining in *Gca*^M-KO^ or *Gca*^flox/flox^ mice fed with MCD diet for 8 weeks (n=6 per group), and quantified as numbers of positive cells per high power field (HPF) (200×). Scale bar, 50 μm.
13. Serum AST and ALT levels in *Gca*^M-KO^ or *Gca*^flox/flox^ mice fed with MCD diet or NCD diet for 8 weeks (n=6 per group).

Data were shown as mean ± SEM. Statistical analysis was assessed by two-sided Student’s t test(B-M). *p<0.05, **p<0.01, ***p<0.001.


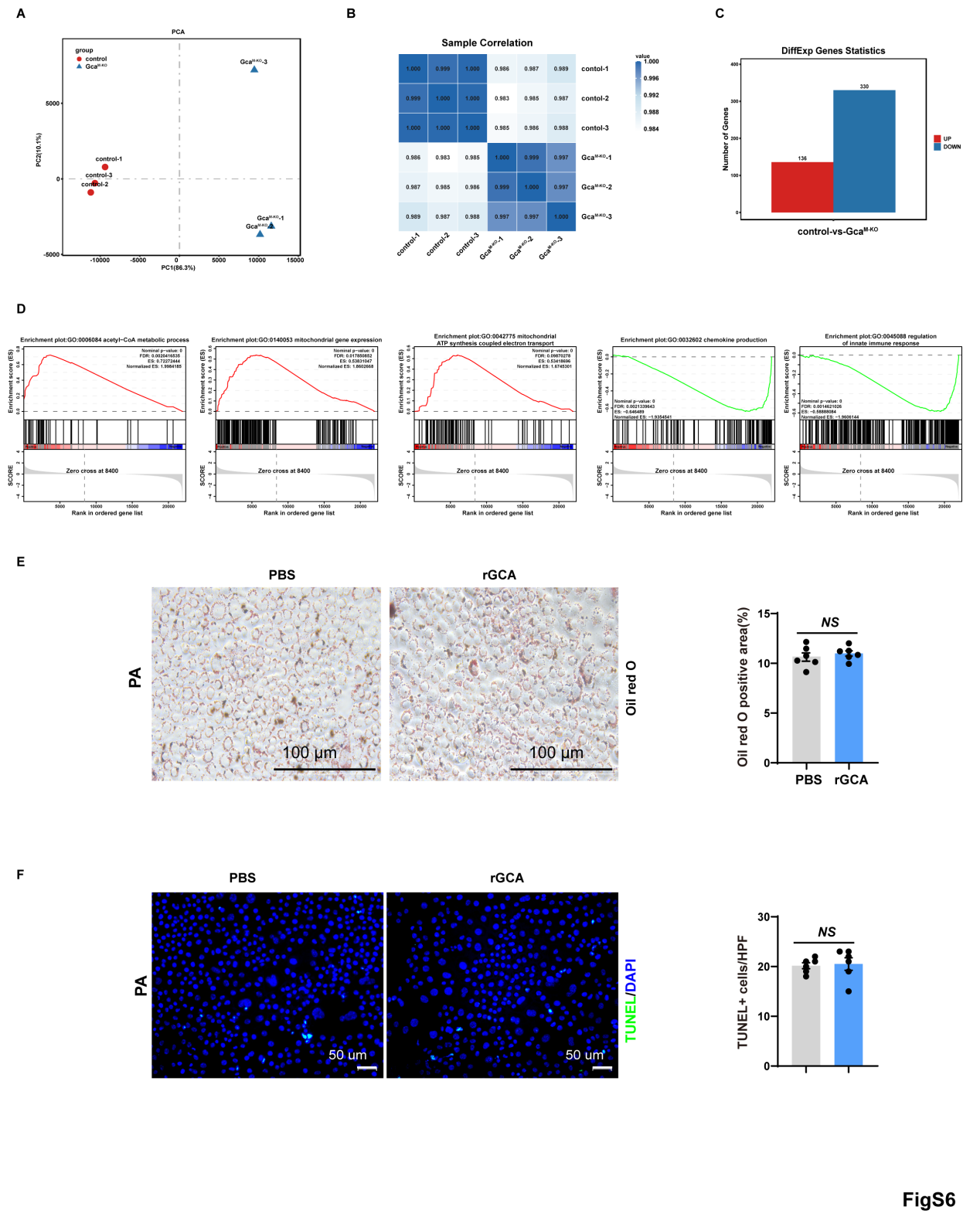


**Figure S6. Mechanistic insights into the protective effect of myeloid *Gca* depletion on MASH**

1. Comparative transcriptome analysis of RNA sequencing (RNA-Seq) in the liver tissues of HFFC diet-fed (24 weeks) *Gca*^flox/flox^ and *Gca*^M-KO^ mice (n=3 per group).
2. Sample correlation of RNA sequencing (RNA-Seq) in the liver tissues of HFFC diet-fed (24 weeks) *Gca*^flox/flox^ and *Gca*^M-KO^ mice (n=3 per group).
3. Histogram showing differentially expressed genes (log_2_(fold change) > 2, FDR(P_adj_) < 0.05) in the liver tissues of *Gca*^flox/flox^ and *Gca*^M-KO^ mice fed with HFFC diet for 24 weeks (n=3 per group), including 136 up-regulated genes and 330 down-regulated genes.
4. Gene Set Enrichment Analysis (GSEA)-GO of differentially expressed genes in *Gca*^flox/flox^ vs. *Gca*^M-KO^ mice fed with HFFC diet for 24 weeks (n=3 per group). Significantly altered pathways are presented.
5. Oil Red O staining of primary hepatocytes in the presense of PA with rGCA/PBS treatment (left) and quantification of Oil Red O staining (right). Scale bar, 100 um.
6. TUNEL staining of primary hepatocytes in the presense of PA with rGCA/PBS treatment and quantified as numbers of positive cells per high power field (HPF) (200×). Scale bar, 50 um.

Data were shown as mean ± SEM.Statistical analysis was assessed by two-sided Student’s t test (E-F). *p<0.05, **p<0.01, ***p<0.001.


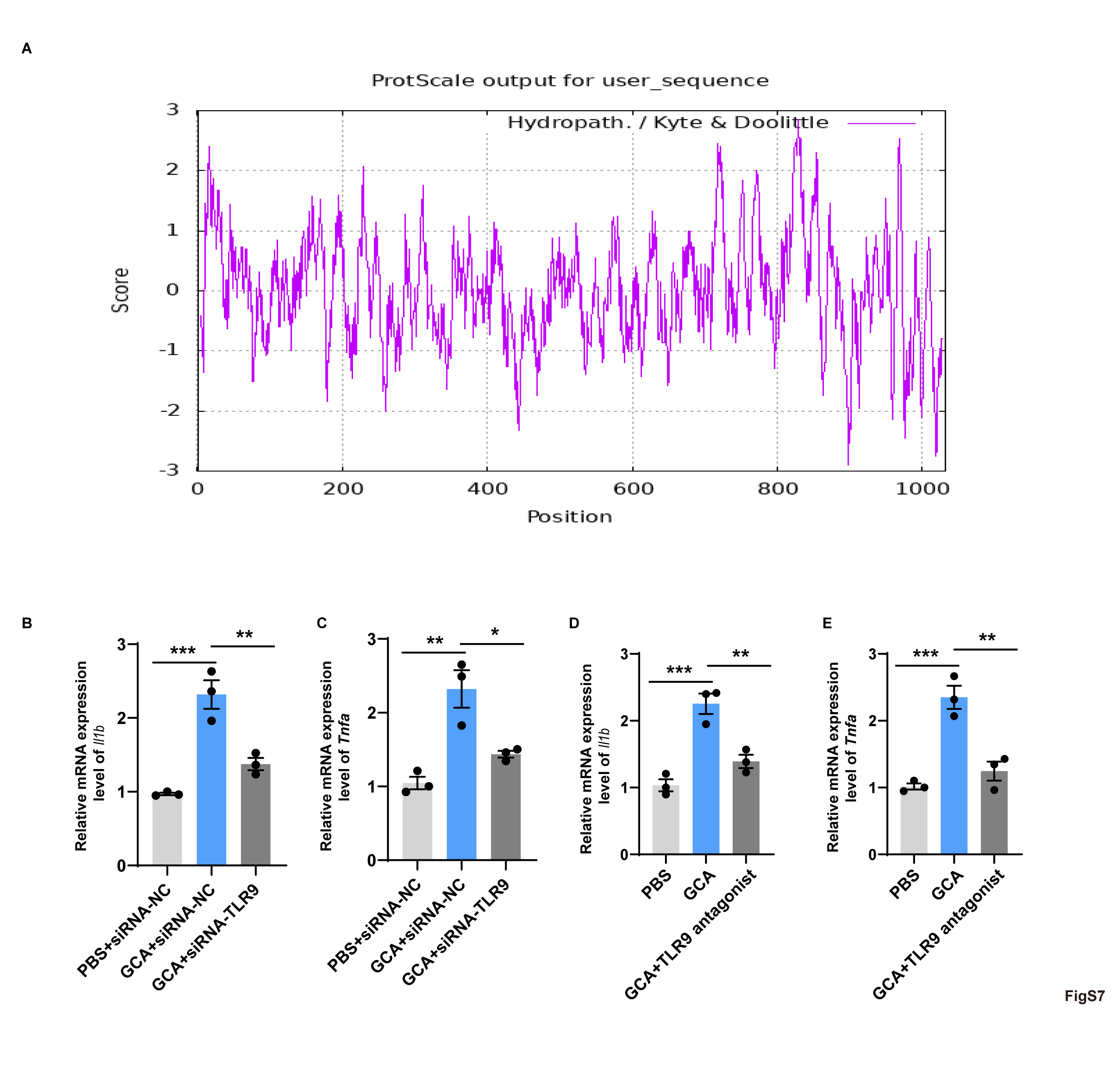


**Figure S7. GCA favors hepatic inflammation via activating TLR9-NFκB signaling in macrophages**

1. Transmembrane helix prediction for TLR9 predicted by ProtScale (<https://web>. expasy.org/protscale/).

(**B-C**) QPCR analysis of *Il1b* (**B**) and *Tnfa* (**C**) in primary hepatic macrophages with addition of PBS+siRNA-NC, GCA+siRNA-NC or GCA+siRNA-TLR9.

(**D-E**) QPCR analysis of *Il1b* (**D**) and *Tnfa* (**E)** in primary hepatic macrophages with addition of PBS, GCA or GCA+TLR9 antagonist.

Data were shown as mean ± SEM. Statistical analysis was assessed by one-way ANOVA with Tukey’s multiple-comparison test (B-E). *p<0.05, **p<0.01, ***p<0.001.


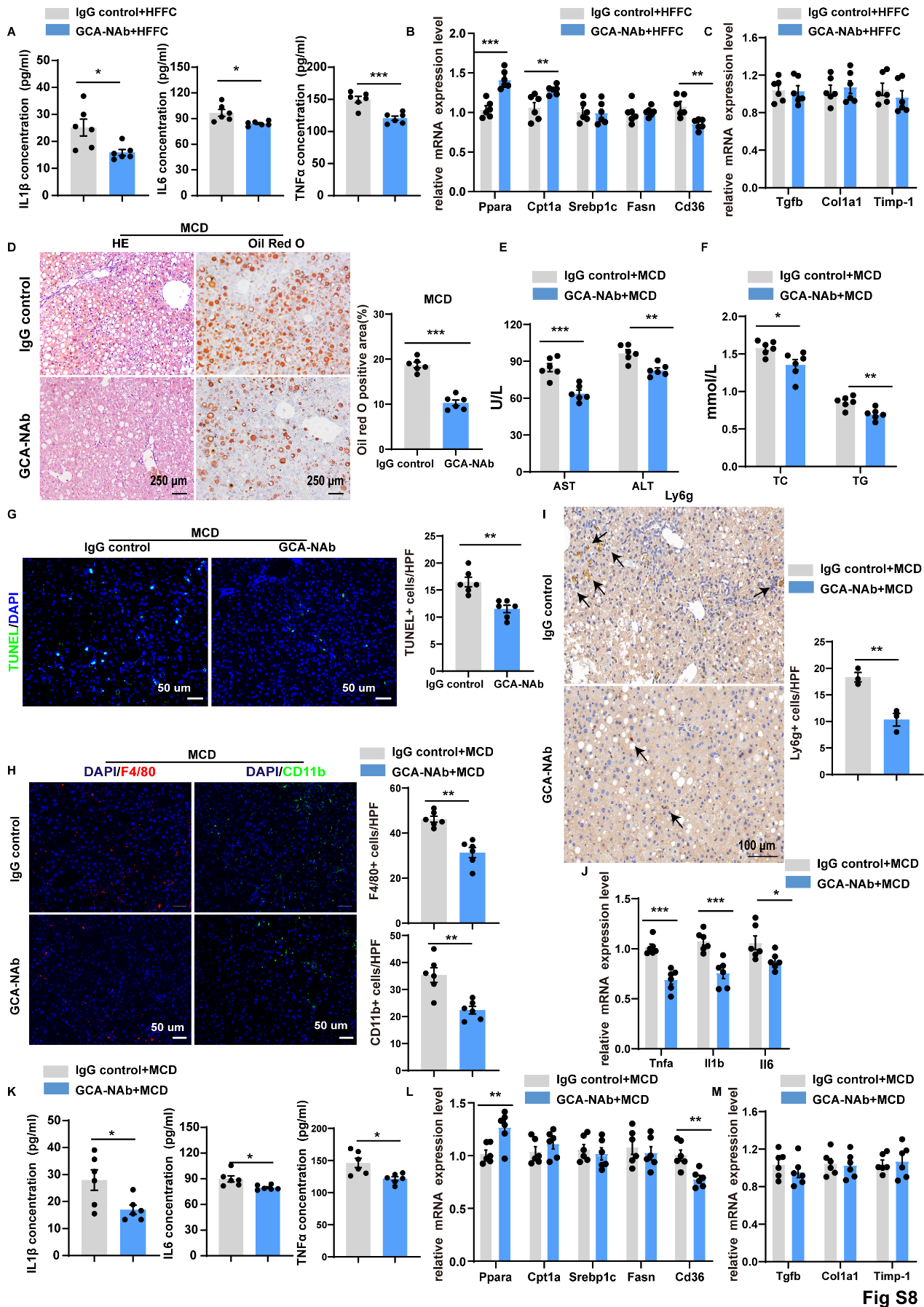


**Fig S8. Anti-GCA antibodies halts the progression of MASH**

1. The concentrations of IL-1β，IL-6 and TNF-α in the serum were measured using ELISA in HFFC diet induced MASH mice treated with LgG or GCA-NAb (n=6 per group).
2. Relative mRNA levels of genes related to fatty acid metabolism in the livers of HFFC diet induced MASH mice treated with LgG or GCA-NAb (n=6 per group).
3. The expression of fibrosis related genes in liver tissues of HFFC diet induced MASH mice treated with LgG or GCA-NAb (n=6 per group).
4. Representative images of HE and Oil Red O staining of liver sections in MCD diet induced MASH mice treated with LgG or GCA-NAb (n=6 per group), and Oil Red O staining quantification in MCD diet induced MASH mice treated with IgG or GCA-NAb (n=6 per group). The Oil Red O staining positive area was quantified by IPP. Scale bar, 250 μm.
5. Serum AST and ALT levels in MCD diet induced MASH mice treated with IgG or GCA-NAb (n=6 per group).
6. Serum TG and TC levels in MCD diet induced MASH mice treated with IgG or GCA-NAb (n=6 per group).
7. TUNEL (green) staining in MCD diet induced MASH mice treated with IgG or GCA-NAb (n=6 per group) (left).TUNEL staining were quantified as numbers of positive cells per high power field (HPF) (200×)(right). Scale bars: 50 μm.
8. Representative immunofluorescence staining showing the expression of F4/80 (red) and CD11b (green) in the liver of MCD diet induced MASH mice treated with IgG or GCA-NAb (n=6 per group) and quantified as numbers of positive cells per high power field (HPF) (200×). Scale bars: 50 μm.
9. Ly6g were detected by immunohistochemistry in the liver of MCD diet induced MASH mice treated with IgG or GCA-NAb (n=6 per group) and quantified as numbers of positive cells per high power field (HPF) (200×). Scale bar, 250 μm.
10. Messenger RNA (mRNA) expression of *Tnfa*, *Il1b* and *Il6* was quantified in liver tissues from MCD diet induced MASH mice treated with IgG or GCA-NAb (n=6 per group).
11. The concentrations of IL-1β, IL-6 and TNF-α in the serum were measured using ELISA in MCD diet induced MASH mice treated with LgG or GCA-NAb (n=6 per group).
12. Relative mRNA levels of genes related to fatty acid metabolism in the livers of MCD diet induced MASH mice treated with LgG or GCA-NAb (n=6 per group).
13. The expression of fibrosis related genes in liver tissues of MCD diet induced MASH mice treated with LgG or GCA-NAb (n=6 per group).

Data were shown as mean ± SEM. Statistical analysis was assessed by two-sided Student’s t test(A-M). *p<0.05, **p<0.01, ***p<0.001.

**Table S1 Characteristics of the Study Population**

Data are presented as mean±SEM. BMI, Body Mass Index; ALT, Alanine aminotransferase; AST, Aspartate aminotransferase; NAS, NAFLD Activity Score.

**Table S2 Nucleotide sequences of primers used for quantitative RT-PCR detection in mouse**

| Trait | Patients with hepatic hemangioma (n=14) | Patients with MASH (n=40) |
| --- | --- | --- |
| Age (years) | 34.92±1.89 | 31.34±7.27 |
| Male/female (n) | 8/6 | 16/24 |
| BMI (kg/m^2^) | 20.19±1.69 | 29.04±0.93 |
| ALT (U/L) | 11.6±1.94 | 75.87±17.89 |
| AST(U/L) | 20.93±3.01 | 57014±12.08 |
| Cholesterol (mmol/l) | 4.42±0.56 | 5.94±0.76 |
| Triglycerides (mmol/l) | 1.45±0.12 | 3.03±1.14 |
| NAS | - | 5.50±0.88 |

| Gene(mouse) | sequence (5' to 3') | |
| --- | --- | --- |
|  | Forward | Reverse |
| *Tgfb* | CGCCATCTATGAGAAAACCAA | GAGTTCCACATGTTGCTCCA |
| *Col1a1* | GCTCCTCTTAGGGGCCACT | CCACGTCTCACCATTGGGG |
| *Timp-1* | GCAACTCGGACCTGGTCATAA | CGGCCCGTGATGAGAAACT |
| *Il6* | GCCCATCCTCTGTGACTCAT | CAGAATTGCCATTGCACAAC |
| *Il1b* | CCCTGCAGCTGGAGAGTGTGGA | CTGAGCGACCTGTCTTGGCCG |
| *Tnfα* | AAGCCTGTAGCCCACGTCGTA | GGCACCACTAGTTGGTTGTCTTTG |
| *Ppara* | GAGAAGTTGCAGGAGGGGATTGTG | AAGACTACCTGCTACCGAAATGGG |
| *Cpt1a* | ATCGTGGTGGTGGGTGTGATAT | ACGCCACTCACGATGTTCTTC |
| *Srebp1c* | TTGGCCACAGTACCTTTGGTT | CTGAGCCTAGGGCCTTGCT |
| *Fasn* | TGGGTTCTAGCCAGCAGAGT | TACCACCAGAGACCGTTATGC |
| *Cd36* | CCTGCAAATGTCAGAGGAAA | GCGACATGATTAATGGCACA |
| *Gca* | GGGGCGTTTGGAAACTTCAG | AGGGGAGTAGCTGTCAGAATAAC |
